# Predicting Breast Cancer Gene Expression Signature by Applying Deep Convolutional Neural Networks From Unannotated Pathological Images

**DOI:** 10.3389/fonc.2021.769447

**Published:** 2021-12-01

**Authors:** Nam Nhut Phan, Chi-Cheng Huang, Ling-Ming Tseng, Eric Y. Chuang

**Affiliations:** ^1^ Bioinformatics Program, Taiwan International Graduate Program, Institute of Information Science, Academia Sinica, Taipei, Taiwan; ^2^ Graduate Institute of Biomedical Electronics and Bioinformatics, National Taiwan University, Taipei, Taiwan; ^3^ Bioinformatics and Biostatistics Core, Centre of Genomic and Precision Medicine, National Taiwan University, Taipei, Taiwan; ^4^ Comprehensive Breast Health Center, Taipei Veterans General Hospital, Taipei, Taiwan; ^5^ Institute of Epidemiology and Preventive Medicine, College of Public Health, National Taiwan University, Taipei, Taiwan; ^6^ School of Medicine, College of Medicine, National Yang Ming Chiao Tung University, Taipei, Taiwan; ^7^ Master Program for Biomedical Engineering, China Medical University, Taichung, Taiwan

**Keywords:** deep learning, convolutional neural networks, breast cancer intrinsic subtypes, pathology, whole slide image, PAM50

## Abstract

We proposed a highly versatile two-step transfer learning pipeline for predicting the gene signature defining the intrinsic breast cancer subtypes using unannotated pathological images. Deciphering breast cancer molecular subtypes by deep learning approaches could provide a convenient and efficient method for the diagnosis of breast cancer patients. It could reduce costs associated with transcriptional profiling and subtyping discrepancy between IHC assays and mRNA expression. Four pretrained models such as VGG16, ResNet50, ResNet101, and Xception were trained with our in-house pathological images from breast cancer patient with recurrent status in the first transfer learning step and TCGA-BRCA dataset for the second transfer learning step. Furthermore, we also trained ResNet101 model with weight from ImageNet for comparison to the aforementioned models. The two-step deep learning models showed promising classification results of the four breast cancer intrinsic subtypes with accuracy ranging from 0.68 (ResNet50) to 0.78 (ResNet101) in both validation and testing sets. Additionally, the overall accuracy of slide-wise prediction showed even higher average accuracy of 0.913 with ResNet101 model. The micro- and macro-average area under the curve (AUC) for these models ranged from 0.88 (ResNet50) to 0.94 (ResNet101), whereas ResNet101_imgnet weighted with ImageNet archived an AUC of 0.92. We also show the deep learning model prediction performance is significantly improved relatively to the common Genefu tool for breast cancer classification. Our study demonstrated the capability of deep learning models to classify breast cancer intrinsic subtypes without the region of interest annotation, which will facilitate the clinical applicability of the proposed models.

## Introduction

Breast cancer is the most common female malignancy in Taiwan, and treatment outcomes have improved enormously in the past decade, attributed to the wide application of screening mammography (early detection at the preclinical stage) and advances in systemic therapy. The use of adjuvant therapy is determined by immunohistochemical (IHC) parameters such as estrogen receptor (ER), progesterone receptor (PR), and human epidermal growth factor receptor II (HER2) status. These factors not only determine which systemic therapy should be given but also predict treatment responses. These pathological factors, however, fail to provide full explanations regarding prognostic heterogeneity observed within each clinical stratum (1). One-fourth of HER2-overexpressing breast tumors eventually develop resistance to trastuzumab, which is a monoclonal anti-HER2 antibody, and endocrine therapy alone is not sufficient for some high-risk hormone-receptor-positive breast cancers. Therefore, an unmet need remains for breast cancer clinic-pathological subtypes, which may be compensated by gene expression-based molecular subtypes.

In the past two decades, gene expression assays have reclassified breast cancers into molecular subtypes based on whole-transcriptome profiles, such as the “intrinsic subtypes” proposed by the Stanford/University of North Carolina group. Perou et al. filtered 476 intrinsic genes from 65 breast cancers and healthy tissues; four subclasses, namely, basal-like, Erb-B2+, normal breast-like, and luminal epithelial/ER+, were identified through clustering analysis (2, 3). The luminal epithelial/ER+ subtype was further subdivided into luminal A and luminal B types, with the latter exhibiting aggressive tumor behavior and worse survival (4). “Intrinsic” genes were defined as those with the highest pair-wise variations between different patients but with the least variations within the same subject. Distinct generations of intrinsic genes have emerged, and the latest one, prediction analysis of microarray 50 gene set (PAM50), was shown to have prognostic and predictive power independent of conventional IHC factors (5, 6). IHC surrogates for intrinsic subtypes using tumor grade instead of Ki-67 were proposed during the 2011 St. Gallen experts’ panel, which demonstrated that gene expression-based molecular subtypes could be approximated by IHC assays (7). However, there was no pathological morphology-driven predictive algorithm for molecular taxonomy until the era of machine learning.

Artificial intelligence (AI) plays a crucial role in biomedical image analyses and cancer research (8, 9). Certain breast cancers behave aggressively, resulting in increased patient morbidity and poor prognosis (10). There are distinguishable cytological features from certain breast carcinomas such as aggressive variants of hereditary breast cancer, poorly differentiated metaplastic carcinoma (11), and triple-negative breast cancer (12). *BRCA1*-associated breast cancers are commonly poorly differentiated, have “medullary features” (a syncytial growth pattern with pushing margins and lymphocytic responses), and are biologically similar to the basal-like subtype defined by gene expression profiling (13). *BRCA2*-associated breast cancers also tend to be poorly differentiated but are more often ER-positive than *BRCA1* mutant counterparts (14). With the availability of digitized whole-slide images (WSIs), it is possible to develop an assisted deep learning based–tool for classification of breast cancer subtypes using these images.

A machine learning algorithm can be applied to histopathological images of breast cancer specimens to see if it can pick out distinguishing features (14). In breast cancer research, there are several gene expression panels to classify and/or predict subtypes, outcomes, or patient survival such as PAM50, which used mRNA expression values of 50 preselected genes (15), while MammaPrint using 70-gene signatures (16), BluePrint using 80-gene signatures (17), and Oncotype DX using 21-gene signatures (18). Recently, there is a growing number of literatures regarding the applications of machine learning and deep learning to predict the mRNA gene expression values using hematoxylin and eosin staining (19) or (synthetic) gene signature prediction using RNA sequencing and clinical information (20). The capacity in predicting gene expression signature taxonomy is extremely important due to the high correlation between these gene panels and patient outcomes or subtypes. The clinical significance of using a low-cost, rapid, and minimal invasive data source such as histopathological WSIs in predicting gene expression signature-based subtype cannot be overemphasized and has been successfully demonstrated (21–23).

Cancer, particularly breast cancer, is a heterogenous disease. Each cancer type might require a specific deep learning model to successfully predict the targets such as subtypes or outcomes stratification. Consequently, it would be of great interest to clinicians if gene expression-defined molecular subtypes could be approximated by computational pattern recognition. In the current study, we developed a complete pipeline using pathological images without region of interest annotation to predict breast cancer PAM50 subtypes. This pipeline comprised a two-step transfer learning technique using four state-of-the-art deep learning architectures, namely, ResNet50 (24), ResNet101 (24), Xception (25), and VGG16 (26). The two-step transfer learning reduced training time and significantly improved the model prediction capacity in both patch-wise and slide-wise approaches.

## Materials and Methods

**Figure 1:**
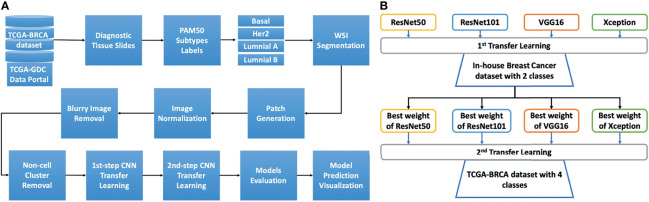
The study design and details of the two-step transfer learning process with pretrained models. **(A)** The overall analysis pipeline. The diagnostic tissue slides of the TCGA-BRCA dataset were downloaded from the TCGA-GDC data portal. Afterward, to retrieve the PAM50 information for each patient, we used the information from the Cancer Genome Atlas Network study, and the cancer subtypes from patients were matched to the image slides for basal, HER2, luminal A, and luminal B These whole slides were then processed for segmentation to select the tiles (patches) with tissue content over 90%. The tiles were then normalized using the Macenko algorithm. Next, blurry tiles and non-cell cluster tiles were removed using Laplacian algorithm. The remaining tiles were then used for two-step transfer learning training. The trained models were then evaluated with the validation set for model selection. The model prediction was visualized using Grad-CAM techniques. **(B)** The two-step transfer learning scheme. Four models, namely, ResNet50, ResNet101, VGG16, and Xception, were used to develop the two-step transfer learning framework. The first step transfer learning was done with weights from the ImageNet dataset for the aforementioned models. These models were trained with our in-house dataset of breast cancer comprising two classes of low and high risk of recurrence. The highest accuracy weights from each model were then used for the second step of transfer learning with the TCGA-BRCA dataset, which includes four classes (subtypes basal, HER2, luminal A, and luminal B).

Overall analytical pipeline is shown in **Figure 1A**, and we used patch-based approaches for model training, in which WSIs were split into thousands of smaller images. Regarding molecular subtypes, 388 WSIs from TCGA-BRCA dataset (https://portal.gdc.cancer.gov) were used to classify patients based on mRNA expression profiles. The PAM50 molecular subtypes of each patient was retrieved from the original publication (27). Additionally, we used an in-house dataset containing 294,591 patches from 233 WSIs of 133 breast cancer patients with known recurrence status (high and low risk) for the first step transfer learning. These WSIs were from Taiwanese breast cancers.

### Patch Generation

A total of 388 WSIs with distinct molecular subtypes of breast cancer underwent patch generation with the PyHIST tool (28). The WSIs of each subtypes are 81, 34, 187, and 86 for Basal, HER2, Luminal A and luminal B, respectively. The patches from each WSI were generated at the highest resolution (40X magnification). The graph method was based on the Otsu algorithm to select images with full tissue coverage. Patches (512x512 pixel) with less than 90% tissue coverage were removed. Tile-crossed image down-sampling and mask down-sampling were set at default values, with a down-sampling factor of 16.

### Images Normalization

The patches generated from PyHIST tool were then normalized for hematoxylin and eosin staining using the Macenko method (29). The original python script was obtained and modified from https://github.com/schaugf/HEnorm_python.

### Blurry Image and Non-Cell Cluster Image Removal

We applied the Laplacian algorithm to remove blurry images using a customized script that calculated variance thresholding for blurry ones. To obtain the suitable kernel size of Laplacian operator, we tried different kernel size from 3x3 pixels to 15x15 pixels. 13x13 pixels was selected as the most suitable kernel size with variance threshold >1e-15 and <1e-14. This process was done using OpenCV tool (30). After blurry and pixelated images were removed, we applied the same procedure to remove images that contained no cells and/or only contained extracellular matrix and cytoplasm, with a threshold of variance >1e24 and kernel size = 21×21.

### General Design for Two-Step Transfer Learning

The concept of the two-step transfer learning is displayed in **Figure 1B**. The general design of two-step transfer learning techniques took advantage of state-of-the-art pretrained models such as ResNet50, ResNet101 (24), Xception (25), and VGG16 (26) as potential useful feature extractors. The first step of transfer learning used the weights of these pretrained models on the ImageNet dataset (31), which is commonly known as a benchmark dataset for deep learning model performance evaluation. These models were trained on our in-house dataset of breast cancer WSIs and took advantage of transfer learning for TCGA-BRCA dataset, which contained similar features for the second step. The two-step process improved model performance and saved training time compared to one-step transfer learning. Finally, gradient-weighted class activation mapping (Grad-CAM) (32) was used for model prediction visualization.

### Transfer Learning Procedure

The first step of transfer learning with ResNet50, ResNet101, Xception, and VGG16 was carried out. The top layers of these models were not included in the feature extraction process, and only convolutional layers were used with weights from ImageNet. These final convolutional layers were connected with a flattened layer and another batch-normalization layer. This layer was then connected to another two fully connected layers of 1,024 neurons and 256 neurons with rectified linear unit (ReLU) activation. This fully connected layer was then linked to the final hidden layer using the sigmoid activation function with one neuron. These models were trained with 50% of our in-house dataset for 50 iterations. The best weight from each of the models was saved using checkpoint and used for second step transfer learning using TCGA-BRCA dataset.

The second step of transfer learning was performed using the same concept as the first step; however, instead of using weights from ImageNet, we loaded the models with the weights obtained from the first step. The last convolutional layers of the loaded models were flattened connected to another two dense layers with 1,024 (D1 layer) and 256 neurons (D2) each. The D2 layer was then connected to the final dense (D3) layer with four neurons, representing the four subtypes of breast cancer. In addition to the four aforementioned models, we trained one more model with ResNet101 using only Imagenet weight for comparing it with the above four models. The model is named ResNet101_imgnet.

### Hyperparameters for Model Training

In total, 1,833,889 patches from TCGA-BRCA dataset were divided into 70% (1,277,407), 5% (99,269), and 25% (457,213) for model training, validation, and testing sets, respectively. The number of images for each cancer subtype is shown in **Table 1**. The testing data were only used when the training and validation procedures were completed to prevent information leaking to the network as well as for the best model evaluation.

**Table 1:** Training, validation, and testing sets for each subtype of breast cancer in the TCGA-BRCA dataset.

Subtypes	Training	Validation	Testing
Basal	280,663	19,990	99,849
HER2	105,295	16,041	40,127
Luminal A	580,404	41,184	206,244
Luminal B	311,045	22,054	110,993
Total	1,277,407	99,269	457,213

We used adaptive moment estimation (ADAM) as the optimizer with a learning rate of 1e^−5^ together with a decay rate of 1e^-5^/50 for 50 epochs for the in-house dataset and 1e^-5^/20 for 20 epochs for the TCGA-BRCA dataset, with the batch size set to 64. Kernel was used with the ReLU function, with the kernel initializer set to “he_uniform”. The Kernel regularizer was used with L2 at 0.0001. The final dense layer was used with the softmax function for final class decision. We used binary cross-entropy and categorical cross-entropy to calculate the loss of accuracy in our model prediction and monitor the training process by binary accuracy and categorial accuracy metrics for the first and second steps of transfer learning, respectively.

Image_datagenerator (ID) was used to fit the data for model training. The ID setup for training data was as follows (rescale = 1/255, rotation_range = 20, zoom_range = 0.05, width_shift_range = 0.1, height_shift = 0.1, shear_range = 0.05, horizontal_flip = True, vertical_flip = True, fill_mode = “nearest”, target_size = (128,128)). The ID for validation and testing was set up with only rescale = 1/255. We used a flow_from_directory model to fit the data to the model, with class_mode = “binary” for the first step of transfer learning and class_mode = “categorical” for the second step of transfer learning with color_mode = “rgb”. Due to the class imbalance with both in-house and TCGA-BRCA datasets, we applied a class_weight term for each class using a standard tensorflow protocol as displayed in formula (1):

W_class(i) = (1/Σ(i))*(Σimages)/N, (1)

where W_class(i) was the weight of class i, Σ(i) was the total number of samples of class i, Σimages was the total number of images of all classes, and N was the number of classes. Model checkpoint and early-stopping techniques were also applied during the training process to preserve the best weight and stop the training process if validation accuracy failed to improve in 5 epochs.

### Model Performance’s Evaluation Metrics

After models had been trained/validated with the two-step transfer learning procedure, we tested the model performance with an independent external testing set. The classification report from scikit learn (33) was used to calculate and display the final classifications of each breast cancer molecular subtype with precision, recall, and F1 score. Additionally, normalized confusion matrix and receiver operating characteristic/area under the curve (ROC-AUC) with micro- and macro-average were used to evaluate model performance. All plots were done using sckikit-learn library (33).

All training was done with Tensorflow version 2.3.0 (Google Inc., Mountain View, CA, USA). The hardware system contained 2 GeForce RTX 2080 Ti GPUs and 128 GB RAM.

### Grad-CAM for Model Visualization

Grad-CAM is the common approach used to visualize how the deep learning model made its decision by tracing back the gradient in the last convolution layer (32). After training our model with WSIs data and obtaining the final optimal weight file, we used this weight to obtain the Grad-CAM visualization with the last convolutional layer in our model. The last convolutions of VGG16, ResNet50, ResNet101, and Xception were “block_conv3”, “conv5_block3_out”, “conv_block3_2_relu”, and “conv2d_3”, respectively. Heatmap images and superimposed images, i.e., overlays of original and heatmap images, were processed with OpenCV (30) and matplotlib (34) library.

### PAM50 Classification With Gene Expression

To compare classifications determined by our deep learning models with the gold standard PAM50 classification using mRNA expression, we used expression profiles of breast cancer patients from TCGA-BRCA downloaded from the University of California Santa Cruz Xena database (https://xenabrowser.net/datapages/). The mRNA expression data were then transformed by median-center normalization and the prediction performed with the Genefu package (35) (ver 2.22.1) in R studio version 1.1.1335 with R version 4.0.3. The “pam50.robust” model was used for subtype prediction to ensure the best concordance with traditional clinical parameters according to the Genefu documentation. The prediction probability of each breast cancer subtype was then exported to a text file by the function “subtype.proba” for comparison. To compare the mean of probability between genefu prediction and deep learning models, we performed Wilcoxon signed rank test with default wilcoxon function in R version 4.0.3.

## Results

### Models’ Training and Validation Performance

Training with the VGG16, ResNet50, Res101, and Xception models achieved 0.73, 0.68, 0.78, and 0.77 classification accuracy with the testing set, respectively. Apart from the accuracy metric, other model evaluation metrics were also calculated for the validation and testing sets, such as the weighted precision, weighted recall, and weighted F1 score (**Table 2**). The lowest F1 score of 0.68 was from the ResNet50 model, and the highest of 0.78 was from the ResNet101 model, whereas the accuracy of the ResNet101_imgnet weights achieved 0.74 accuracy. The Xception and VGG16 models’ weighted F1 scores were 0.77 and 0.73, respectively (**Table 2**).

**Table 2:** Patch-wise model performance on the validation and testing sets.

Models/Metrics		Validation/Testing results			
		Basal	HER2	Luminal A	Luminal B
ResNet50	Precision	0.53/0.56	0.81/0.69	0.76/0.78	0.62/0.65
	Recall	0.76/0.76	0.53/0.54	0.72/0.73	0.58/0.58
	F1-score	0.62/0.64	0.64/0.61	0.74/0.75	0.60/0.61
ResNet101	Precision	0.70/0.72	0.81/0.68	0.84/0.86	0.73/0.75
	Recall	0.80/0.79	0.81/0.82	0.78/0.78	0.74/0.74
	F1-score	0.75/0.76	0.81/0.74	0.81/0.82	0.74/0.74
ResNet101_imgnet	Precision	0.65/0.68	0.76/0.61	0.83/0.84	0.65/0.67
	Recall	0.80/0.80	0.74/0.74	0.73/0.73	0.69/0.69
	F1-score	0.72/0.74	0.75/0.67	0.77/0.78	0.67/0.68
VGG16	Precision	0.58/0.61	0.76/0.62	0.86/0.87	0.69/0.72
	Recall	0.85/0.85	0.71/0.72	0.72/0.72	0.64/0.64
	F1-score	0.69/0.71	0.73/0.66	0.78/0.79	0.67/0.78
Xception	Precision	0.62/0.64	0.82/0.70	0.85/0.86	0.77/0.78
	Recall	0.86/0.86	0.75/0.75	0.78/0.78	0.66/0.66
	F1-score	0.72/0.73	0.78/0.73	0.81/0.82	0.71/0.72

**Figure 2:**
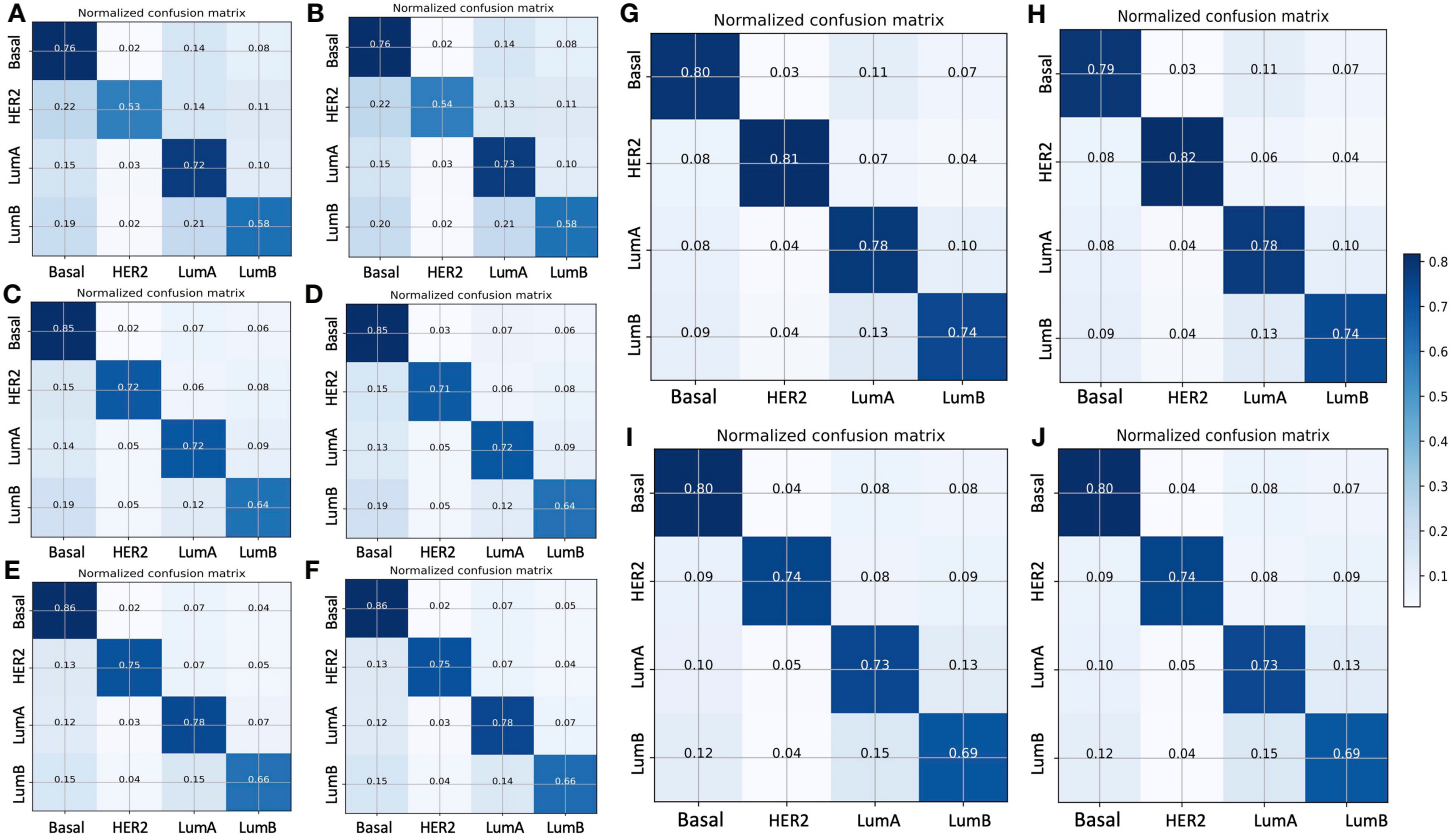
Normalized confusion matrix of five models with the validation (left panels) and testing (right panels) sets. **(A, B)** ResNet50 model, **(C, D)** VGG16 model, **(E, F)** Xception model, **(G, H)** ResNet101 model, **(I, J)** ResNet101_imgnet model. True and predicted subtype classifications are shown on the y- and x-axes, respectively, such that the correct predictions are shown on the diagonal from the top left to the bottom right of each matrix. The blue gradient color represents the model accuracy for detecting each subtype. The darker the blue color, the better the model performance.

**Figure 2** displays the normalized confusion matrix of each model on the validation and testing sets. In general, all models had almost identical performance on the validation and testing sets. The lowest-performing model was ResNet50, and the highest-performing model was ResNet101. For instance, ResNet50 (**Figures 2A, B**) had 24% wrong predictions for the basal subtype and 2%, 14%, and 8% wrong predictions for the HER2-enriched, luminal A, and luminal B subtypes, respectively. The VGG16 (**Figures 2C, D**) and Xception (**Figures 2E, F**) models had better prediction for the basal-like subtype, with 15% and 14% wrong predictions, respectively. While the ResNet101 model (**Figures 2G, H**) had only 21% wrong predictions of the basal-like subtype, the ResNet101_imgnet model (**Figures 2I, J**) had 20% wrong predictions of the same subtype. The poorest performance was with the HER2-enriched subtype, with the ResNet50 model at a 47% error rate on the testing set. The best performance on luminal A classification was from the ResNet101 and Xception models, both of which reported a 78% correct prediction rate. Furthermore, ResNet101 was the best predictor for the luminal B subtype, with 74% correct predictions compared to the second-best model (Resnet101_imgnet), which achieved 69% correct predictions.

**Figure 3:**
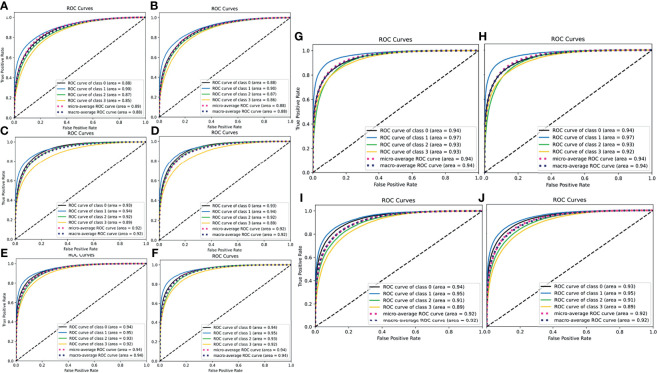
ROC-AUC curves for the true positive and false positive rate of all five models (left panels for validation set, right panels for testing set). **(A, B)** ResNet50 model, **(C, D)** VGG16 model, **(E, F)** Xception model, **(G, H)** ResNet101 model, **(I, J)** ResNet101_imgnet model. In the individual legends, classes 0, 1, 2, and 3 represent basal-like, HER2-enriched, luminal A, and luminal B subtypes, respectively. The higher the AUC, the better the model in detecting true positives and producing fewer false positives.

To further evaluate the true positive rate and false positive rate of each model on the validation and testing sets, we plotted the ROC-AUC curve of each model, as well as the micro- and macro-average ROC curves of all classes (**Figure 3**). The highest micro-average AUC was 0.94, which belonged to the Xception model (**Figures 3E, F**) and the ResNet101 model (**Figures 3G, H**), whereas the lowest belonged to the ResNet50 model (micro-average AUC=0.89) (**Figures 3A, B**). The VGG16 model had an AUC of 0.92 (**Figures 3C, D**), which was equal to that of the ResNet101_imgnet model. Together with the confusion matrices and ROC curve metrics, we also used precision, recall, and F1 score for evaluating all the models’ performance in the validation and testing sets. For the patch-wise approach, the precision, recall, and F1 score of the basal-like, HER2-enriched, luminal A, and luminal B subtypes are displayed in **Table 2**. Overall, the F1 scores on the testing set were nearly identical to those of the evaluation. The highest model performance with the testing set was still achieved by ResNet101, with F1 scores of 0.76, 0.74, 0.82, and 0.74 for basal-like, HER2-enriched, luminal A, and luminal B subtypes, respectively.

### Models’ Performance on the Testing Set With a Slide-Wise Approach

**Figure 4:**
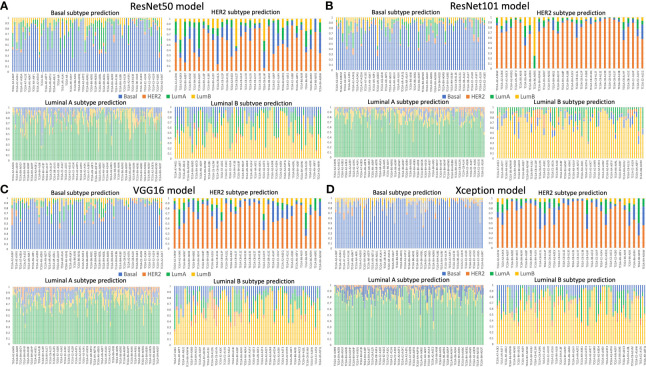
Slide-wise subtype prediction using the testing set. **(A)** ResNet50 model, **(B)** ResNet101 model, **(C)** VGG16 model, **(D)** Xception model. Basal, HER2, luminal A, and luminal B subtypes are represented in blue, orange, green, and yellow bars, respectively. A threshold of 0.5 was used to select the correct prediction subtype from the whole slide image of each patient. The prediction probability of each subtype was aggregated from the patches of the same patient.

We validated the model performance with a testing set of 457,213 patches comprising four subtypes as shown in **Table 1**. In addition to the patch-wise approach described above, we used a slide-wise approach because each patient would have more than one WSI for final subtype assessment in routine clinical practice. The slide-wise approach had the highest confidence, with 0.913 overall accuracy across all four subtypes using the ResNet101 model. The slide-wise method was better for clinical use because it provided higher confidence in the model prediction based on the PAM50 signature in clinical applications. The slide-wise results of all four models for each subtype are displayed in **Figures 4A–D**. To maintain comparability with Genefu prediction probability, the threshold was determined as 0.5 for subtype classification of each patient (**Table S1**). Each model’s performance on slide-wise analysis is detailed in **Table 3**. The ResNet101 model’s accuracy was 0.89, 0.9375, 0.969, and 0.857 for the basal-like, HER2-enriched, luminal A, and luminal B subtypes, respectively. The second-highest model performance was the Xception model with an average accuracy of 0.856. ResNet50 had the poorest performance with an average accuracy of 0.698, whereas the VGG16 and ResNet101_imgnet models had average accuracies of 0.851 and 0.834, respectively. Additionally, weighted average of precision, recall, and F1 score of each model are displayed in **Table 4** together with the results from previous study performed by Jaber et al. (36). The best model performance (ResNet101) reached 0.78 for all accuracy, precision, recall, and F1 score. The poorest model performance (ResNet50) with 0.68, 0.69, 0.68, and 0.68 for the same set of metrics.

**Table 3:** Slide-wise model performance on the testing set.

Model	Accuracy				
	Basal	HER2	Luminal A	Luminal B	Average
ResNet50	0.767	0.563	0.905	0.557	0.698
ResNet101	**0.890**	**0.9375**	**0.969**	**0.857**	**0.913**
ResNet101_imgnet	0.904	0.7812	0.924	0.729	0.834
VGG16	0.918	0.875	0.955	0.657	0.851
Xception	0.972	0.719	0.975	0.757	0.856

Bold font denotes the best model.

**Table 4:** Patch-wise model performance on the testing set in comparison to a previous study.

Model	Weighted average Accuracy	Weighted average Precision	Weighted average Recall	Weighted average F1 Score
ResNet50	0.68	0.69	0.68	0.68
ResNet101	**0.78**	**0.78**	**0.78**	**0.78**
ResNet101_imgnet	0.73	0.75	0.73	0.74
VGG16	0.73	0.75	0.70	0.73
Xception	0.77	0.78	0.77	0.77
Jaber et al.	0.59–0.66	N/A	N/A	N/A

Bold font denote the best model. N/A: no information available.

### Comparison Between DL Models Prediction and Genefu Prediction

Furthermore, to compare the confidence of model prediction probability from our study, the golden standard Genefu tool, we calculated and showed the mean and standard deviation of deep learning models in comparison to that from Genefu (**Table 5**). Overall, based on Wilcoxon signed rank test results, we found that mean probability in the basal-like subtype was not significantly different from the deep learning model, whereas the other subtypes such as HER2-enriched, luminal A, and luminal B subtypes showed significant difference between these mean probabilities, which means the DL model prediction has significantly improved the prediction probability for each patient compared to the Genefu package.

**Table 5:** Comparison of mean of probability between Genefu method and deep learning models by Wilcoxon signed rank test.

	Basal	HER2	Luminal A	Luminal B
Genefu	0.804 ± 0.202	0.50 ± 0.123	0.668 ± 0.098	0.543 ± 0.183
ResNet50	0.723 ± 0.224 (0.02718)	0.527 ± 0.228 (0.2214)	0.748* ± 0.174 (4.113e-07)	0.561 ± 0.257 (0.6809)
ResNet101	0.783 ± 0.188 (0.2949)	0.796* ± 0.143 (7.577e-06)	0.787* ± 0.134 (2.615e-15)	0.718* ± 0.190 (8.048e-06)
ResNet101_imgnet	0.761 ± 0.179 (0.1612)	0.699* ± 0.225 (0.005995)	0.737* ± 0.148 (5.104e-07)	0.664* ± 0.216 (0.003084)
VGG16	0.811 ± 0.176 (0.4308)	0.668* ± 0.187 (0.02027)	0.729* ± 0.136 (6.228e-06)	0.632* ± 0.216 (0.03663)
Xception	0.836 ± 0.163 (0.163)	0.729* ± 0.195 (0.002992)	0.797* ± 0.133 (3.573e-16)	0.654* ± 0.210 (0.004434)

The values were presented as mean ± SD (p-value). *denotes the significant improvement of deep learning model in comparison to Genefu model (p-value < 0.05).

### Models’ Prediction Visualization

**Figure 5:**
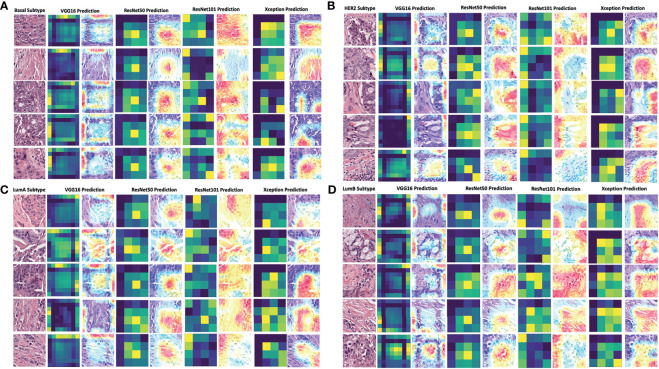
Grad-CAM visualization of deep learning models in four breast cancer subtypes. The visualization of each model’s prediction for the same set of images from five patients with breast cancer of the basal subtype **(A)**, HER2 subtype **(B)**, luminal A subtype **(C)**, and luminal B subtype **(D)**. The heatmap in dark green and yellow colors was plotted together with the merged images of the original and activated heatmaps. The activation levels in the merged images are shown with a gradient of red, yellow, and blue areas, which the models used to make a classification decision. LumA: Luminal A; LumB: Luminal B.

To decode the model learning process, we used Grad-CAM with the last convolution layer to create a heatmap superimposed on the original image. This illustrated how each model learned to distinguish differences among the four breast cancer subtypes (**Figures 5A–D**). It was readily seen that the VGG16 model’s heatmap was highly activated over the edges of the image for all subtypes, whereas the ResNet50 and ResNet101 models’ heatmaps were activated in the middle of the image for all subtypes. The Xception model’s activation was quite different for each subtype. Regardless of the corner or middle activation of these models, they demonstrated all models’ capability and logic in distinguishing cell cluster and non-cell cluster areas.

## Discussion

In the present study, we applied deep learning techniques for breast cancer intrinsic subtypes classification, using pathological images as the only source of input without annotation for tumor areas. ResNet101 achieved the highest performance in both validation and testing sets, with either patch-wise (accuracy of 0.78) or slide-wise (accuracy of 0.913) approach. The heterogeneity of breast cancer was also observed across all four intrinsic subtypes based on the prediction probability distributions across various deep learning models. We believe the comprehensive pipeline in the current study is applicable to more than just a single type of cancer, for which breast cancer is the case; it can be generalized to other types of cancers after a simple change in data labeling. This pipeline can assist pathologists to classify breast cancer subtypes rapidly and has great potential to augment PAM50 subtyping using gene expression data with enhanced concordance for each subtype. The two-step transfer learning is proved to outperform the common transfer learning protocol with weights from Imagenet.

It is widely accepted that different breast cancer molecular subtypes have discrepant prognoses over time. Understanding different recurrence patterns can improve breast cancer care *via* the application of surveillance guidelines, which can identify the most optimistic treatment (37). According to Metzger-Filho et al., basal-like and HER2-enriched cohorts had higher risk of recurrence in the first 4 years after diagnosis. On the other hand, luminal B had a continuously higher hazard of recurrence over the 10-year follow-up compared to luminal A breast cancer (37). Another study by Ribelles et al. reported that luminal A cancer displayed a slow risk increase, reaching its maximum after 3 years and then remaining steady, while luminal B cancer presented most of its relapses during the first 5 years (38). Compared to HER2-overexpressing and triple-negative cancers, ER-positive breast cancers exhibited more mid- to long-term relapses and acquired *ESR1* mutations, resulting in the ligand-independent and constitutive activation of the ER that is believed to play a major role in late recurrence and endocrine therapy resistance (39). Deciphering breast cancer molecular subtypes by deep learning approaches could provide a convenient and efficient method for diagnosis of breast cancer patients. It could reduce costs associated with transcriptional profiling and subtyping discrepancy between IHC assays and mRNA expression. In terms of academic development, this is a novel approach that can lay the foundation for later research on breast cancer taxonomy and precision medicine.

We are approaching an era of AI- and machine learning-aided diagnosis and treatment, and breast pathology imaging may be one of the frontiers. AI has the potential to transform genomics, pathology, and breast oncology, and current deep learning systems are starting to match human performance in reading pathological and morphological features and reducing inter-observer variability (14, 21–23). In the current study, ResNet101 achieved an overall accuracy of 78% in the validation and testing sets, whereas the ResNet50 model achieved 68% accuracy (**Table 4**). In a study performed by Jaber et al., with the region of interest labeled by pathologists, their model accuracy ranged from 0.586 to 0.661 (40). In previous literature, many models have been developed to predict or classify a wide range of diagnostic or therapeutic targets of breast cancer. Cruz-Roa et al. designed a convolutional neural network to detect the location of invasive tumors in whole pathological images. In their study, they used 400 samples for training and validated the model with 200 samples from TCGA database. The model achieved a Dice coefficient of 75.86 and 71.62% positive predictive value and 96.77% negative predictive value relative to manual annotation (41). In another study, Zheng et al. applied the K-means algorithm to classifying benign and malignant lesions. The feature was extracted and trained with a support vector machine (SVM). This model reached 97% accuracy with 10-fold cross-validation on a Wisconsin Diagnostic Breast Cancer dataset (42). Google’s Inception model has been used for identifying cancer subtypes with extensive tumor heterogeneity with accuracy rates of 100, 92, 95, and 69% for various cancer tissues, subtypes, biomarkers, and scores, respectively (43). Another study by Alakwaa et al. used a cohort of 548 cancer patients to train feed-forward networks within a deep learning framework. They found that the deep learning method achieved an AUC of 0.93 in classifying ER+ and ER− breast cancer patients (44).

Furthermore, in an attempt to demonstrate that deep learning models trained on pathological images could be potential tools in assisting pathologists to predict breast cancer using the PAM50 gene signature, we compared the mean of probability between our deep learning models to the commonly used package for PAM50 classification, Genefu (35). Except for the basal-like subtype, means of probability of the remaining three subtypes were significantly improved from the Genefu prediction. These comparisons illustrated that the results from deep learning models with only pathological WSIs as their data source can be used as an initial tool for breast cancer intrinsic subtypes classification.

The potential applications of the current protocol are not only for breast cancer but also can be used for pan-cancer study. Apart from gene expression signature prediction, this pipeline can also be used to predict the overall survival or disease-free survival duration of cancer patients. We have implemented this pipeline with the renal cancer dataset downloaded from TCGA database. The model successfully predicted patients’ survival in different years categories with weighted accuracy of 95.5% (data not shown). Estimating the cancer patient survival from a single source of data for such as pathological images with high resolution facilitates the prediction accuracy and is cost-efficient.

There were some limitations in the current study. The developed protocol for predicting gene expression signatures using pathological images was only tested using two breast cancer datasets. For future prospective studies, this pipeline can be applied for predicting gene expression signatures with enhanced power of evidence by including clinical information into the training data. Depending on the prediction targets, the clinical information such as age, cancer stage, tumor size, and grades could add more features, which might be important for model learning and potentially improve model prediction performance. There exist imbalances among the breast cancer subtypes in the TCGA-BRCA dataset used in this study, which might explain why the model performed well for certain subtypes but not for others. For the intrinsic heterogeneity in other types of cancers, it is recommended to have a balance dataset between subtypes as the amount of data from each subtype might have an effect on the model performance. However, in real-world cancer datasets, the model performance may be highly dependent on the nature of the cancer type and study cohort; therefore, acquiring a balance dataset is a caveat.

## Conclusions

In summary, our study provided a practical deep learning-based pipeline for classifying breast cancer molecular subtypes *via* a two-step transfer learning protocol that used pathological images as the source for training of deep convolutional neural networks. These deep learning models had the potential to assisting pathologists and physicians to classify breast cancer subtypes rapidly with good reliability and accuracy.

## Data Availability Statement

The datasets presented in this study can be found in online repositories. The names of the repository/repositories and accession number(s) can be found below: portal.gdc.cancer.gov/repository/; accession number: TCGA-BRCA.

## Ethics Statement

The studies involving human participants were reviewed and approved by Taipei General Hospital. The patients/participants provided their written informed consent to participate in this study.

## Author Contributions

Conceptualization: NP and EC. Methodology: NP and C-CH. Validation: NP and C-CH. Formal analysis: NP. Investigation: NP and C-CH. Resources: EC and L-MT. Data curation: NP and C-CH. Writing, original draft preparation: NP and C-CH. Writing, review and editing: NP and C-CH. Visualization: NP and C-CH. Supervision: EC and L-MT. Project administration: EC and L-MT. Funding acquisition: EC and L-MT. All authors contributed to the article and approved the submitted version.

## Funding

This work was supported in part by the Center of Genomic and Precision Medicine, National Taiwan University, the Ministry of Science and Technology, Taiwan (Grant No. MOST-110-2634-F-002-044), and the Center for Biotechnology, National Taiwan University, Taiwan (Grant No. GTZ300). This study was also partly funded by the Taipei Veterans General Hospital (Grant No. V110E-005-3).

## Conflict of Interest

The authors declare that the research was conducted in the absence of any commercial or financial relationships that could be construed as a potential conflict of interest.

## Publisher’s Note

All claims expressed in this article are solely those of the authors and do not necessarily represent those of their affiliated organizations, or those of the publisher, the editors and the reviewers. Any product that may be evaluated in this article, or claim that may be made by its manufacturer, is not guaranteed or endorsed by the publisher.
