# Supplementary material for: Predicting Breast Cancer Gene Expression Signature by Applying Deep Convolutional Neural Networks From Unannotated Pathological Images
Source: Front Oncol. 2021 Dec 1;11:769447. doi: 10.3389/fonc.2021.769447 (PMC8673486; doi:10.3389/fonc.2021.769447)
Supplement: Supplementary file 1 [file Table_1.docx]

Table S1. Genefu prediction probability with TCG-BRCA dataset form mRNA expression values.

| Sample ID | Predicted subtype | Probability of each subtype | | | | |
| --- | --- | --- | --- | --- | --- | --- |
|  |  | Basal | Her2 | LumA | LumB | Normal |
| TCGA-A8-A0A7-01 | Her2 | 0 | 0.54456742 | 0.14716091 | 0.30827166 | 0 |
| TCGA-A8-A07G-01 | LumA | 0 | 0 | 0.71673873 | 0 | 0.28326127 |
| TCGA-A8-A08R-01 | Basal | 0.55744975 | 0.2371134 | 0 | 0.20543685 | 0 |
| TCGA-A8-A06Y-01 | LumA | 0 | 0 | 0.72767531 | 0.0331403 | 0.23918439 |
| TCGA-A8-A08B-01 | Her2 | 0 | 0.60329213 | 0 | 0.39670787 | 0 |
| TCGA-A2-A04T-01 | Basal | 1 | 0 | 0 | 0 | 0 |
| TCGA-A2-A0EQ-01 | Her2 | 0 | 0.56191675 | 0 | 0.43808325 | 0 |
| TCGA-A2-A0CQ-01 | LumA | 0 | 0 | 0.81607691 | 0 | 0.18392309 |
| TCGA-BH-A0EB-01 | LumA | 0 | 0 | 0.71012385 | 0 | 0.28987615 |
| TCGA-A2-A04P-01 | Basal | 1 | 0 | 0 | 0 | 0 |
| TCGA-BH-A0E7-01 | LumA | 0 | 0 | 0.72030172 | 0 | 0.27969828 |
| TCGA-A8-A08T-01 | LumA | 0 | 0 | 0.71966382 | 0 | 0.28033619 |
| TCGA-A8-A08G-01 | LumB | 0 | 0.04875526 | 0.25674091 | 0.69450383 | 0 |
| TCGA-A8-A09M-01 | LumB | 0 | 0.03886887 | 0.44463543 | 0.5164957 | 0 |
| TCGA-A8-A0A4-01 | LumA | 0 | 0 | 0.61512554 | 0 | 0.38487446 |
| TCGA-BH-A0BV-01 | LumA | 0 | 0 | 0.66176809 | 0.18754319 | 0.15068872 |
| TCGA-AN-A04D-01 | Basal | 0.8292011 | 0.04118104 | 0 | 0.12961786 | 0 |
| TCGA-A2-A0D2-01 | Basal | 0.83615054 | 0.06990989 | 0 | 0.09393957 | 0 |
| TCGA-B6-A0IC-01 | LumA | 0 | 0 | 0.7635877 | 0.10973944 | 0.12667286 |
| TCGA-A8-A06U-01 | LumA | 0 | 0.14841127 | 0.52174253 | 0.3298462 | 0 |
| TCGA-AN-A0FD-01 | LumA | 0 | 0 | 0.82768339 | 0.05483533 | 0.11748128 |
| TCGA-A7-A0CE-01 | Basal | 0.83048236 | 0 | 0 | 0 | 0.16951764 |
| TCGA-A2-A0CX-01 | Her2 | 0 | 0.65552359 | 0 | 0.34447641 | 0 |
| TCGA-A2-A04Q-01 | Basal | 1 | 0 | 0 | 0 | 0 |
| TCGA-A2-A0CM-01 | Basal | 1 | 0 | 0 | 0 | 0 |
| TCGA-A8-A081-01 | LumB | 0 | 0.47873375 | 0 | 0.52126625 | 0 |
| TCGA-A8-A09A-01 | LumA | 0 | 0 | 0.72238388 | 0 | 0.27761612 |
| TCGA-A8-A09X-01 | Her2 | 0 | 0.54495527 | 0 | 0.45504473 | 0 |
| TCGA-A8-A07W-01 | LumB | 0 | 0.41067842 | 0 | 0.58932158 | 0 |
| TCGA-A2-A0ER-01 | LumB | 0 | 0 | 0.45237344 | 0.54762656 | 0 |
| TCGA-AN-A04A-01 | LumA | 0 | 0 | 0.63236329 | 0 | 0.36763672 |
| TCGA-A8-A06T-01 | LumA | 0 | 0 | 0.78922106 | 0.09709396 | 0.11368498 |
| TCGA-A8-A07Z-01 | LumA | 0 | 0.00741376 | 0.57796704 | 0.4146192 | 0 |
| TCGA-A8-A08X-01 | Her2 | 0.07824854 | 0.54424996 | 0 | 0.3775015 | 0 |
| TCGA-A8-A0AB-01 | LumA | 0 | 0 | 0.61861417 | 0.38138583 | 0 |
| TCGA-BH-A0DZ-01 | Her2 | 0 | 0.47645475 | 0.26149788 | 0.26204737 | 0 |
| TCGA-AN-A0FV-01 | Her2 | 0 | 0.53576163 | 0.33047028 | 0.13376809 | 0 |
| TCGA-B6-A0IG-01 | Her2 | 0 | 0.41717005 | 0.34175362 | 0.24107633 | 0 |
| TCGA-A8-A09I-01 | LumB | 0 | 0.45652339 | 0 | 0.54347661 | 0 |
| TCGA-AN-A0FG-01 | LumB | 0 | 0.33350929 | 0 | 0.66649071 | 0 |
| TCGA-B6-A0I6-01 | Basal | 1 | 0 | 0 | 0 | 0 |
| TCGA-AN-A0FY-01 | LumB | 0 | 0.23360807 | 0.06458359 | 0.70180834 | 0 |
| TCGA-A8-A082-01 | LumB | 0 | 0.22206874 | 0.15105225 | 0.62687901 | 0 |
| TCGA-AO-A0J8-01 | LumA | 0 | 0 | 0.75884407 | 0 | 0.24115593 |
| TCGA-A8-A092-01 | LumB | 0 | 0.4718762 | 0 | 0.5281238 | 0 |
| TCGA-AO-A0J7-01 | LumB | 0 | 0.17452503 | 0.11973742 | 0.70573755 | 0 |
| TCGA-A8-A09V-01 | LumA | 0 | 0 | 0.74047159 | 0.10838082 | 0.15114759 |
| TCGA-A8-A08S-01 | LumA | 0 | 0.18950295 | 0.44578313 | 0.36471392 | 0 |
| TCGA-AO-A0J2-01 | Her2 | 0.15560982 | 0.51856374 | 0 | 0.32582644 | 0 |
| TCGA-AO-A0J6-01 | Basal | 0.77031015 | 0.18831677 | 0 | 0.04137308 | 0 |
| TCGA-BH-A0AY-01 | LumA | 0 | 0.17751171 | 0.49862423 | 0.32386406 | 0 |
| TCGA-A8-A07S-01 | LumB | 0 | 0 | 0.40218772 | 0.59781228 | 0 |
| TCGA-AN-A0FS-01 | LumA | 0 | 0 | 0.69741315 | 0 | 0.30258685 |
| TCGA-B6-A0I5-01 | LumA | 0 | 0 | 0.65998487 | 0 | 0.34001513 |
| TCGA-A8-A083-01 | LumA | 0 | 0 | 0.70170522 | 0 | 0.29829478 |
| TCGA-AN-A049-01 | LumB | 0 | 0.04156818 | 0.46797348 | 0.49045835 | 0 |
| TCGA-AO-A0J9-01 | LumA | 0 | 0 | 0.7691745 | 0.2308255 | 0 |
| TCGA-B6-A0IA-01 | LumA | 0 | 0 | 0.65389642 | 0.34610358 | 0 |
| TCGA-A7-A0DC-01 | LumA | 0 | 0 | 0.68548984 | 0 | 0.31451016 |
| TCGA-AN-A0FL-01 | Basal | 0.53967158 | 0 | 0 | 0 | 0.46032842 |
| TCGA-A8-A07F-01 | LumA | 0 | 0 | 0.7643022 | 0.12758126 | 0.10811654 |
| TCGA-A7-A0CG-01 | LumA | 0 | 0 | 0.64503579 | 0 | 0.35496421 |
| TCGA-BH-A0HQ-01 | LumA | 0 | 0 | 0.70947051 | 0 | 0.29052949 |
| TCGA-AO-A03O-01 | LumB | 0 | 0.25742916 | 0 | 0.74257084 | 0 |
| TCGA-B6-A0IE-01 | LumA | 0 | 0 | 0.63892994 | 0.36107006 | 0 |
| TCGA-AN-A0FN-01 | LumA | 0 | 0 | 0.75041127 | 0 | 0.24958873 |
| TCGA-A8-A06X-01 | LumB | 0 | 0.20303722 | 0 | 0.79696278 | 0 |
| TCGA-A8-A09Z-01 | LumA | 0 | 0.13821241 | 0.65285597 | 0.20893162 | 0 |
| TCGA-A8-A0A9-01 | LumA | 0 | 0 | 0.7141276 | 0.2858724 | 0 |
| TCGA-A8-A09T-01 | LumA | 0 | 0 | 0.76720407 | 0 | 0.23279593 |
| TCGA-AO-A0J5-01 | LumA | 0 | 0.05558719 | 0.79309609 | 0.06740214 | 0.08391459 |
| TCGA-A8-A097-01 | LumB | 0 | 0.39932331 | 0.12062318 | 0.48005351 | 0 |
| TCGA-BH-A0B4-01 | LumA | 0 | 0.18024764 | 0.50423335 | 0.31551901 | 0 |
| TCGA-B6-A0I2-01 | Basal | 1 | 0 | 0 | 0 | 0 |
| TCGA-A8-A06P-01 | LumA | 0 | 0 | 0.70189094 | 0 | 0.29810906 |
| TCGA-A8-A08I-01 | LumB | 0 | 0 | 0.29050752 | 0.70949248 | 0 |
| TCGA-A8-A084-01 | LumB | 0 | 0.24665303 | 0.1736919 | 0.57965507 | 0 |
| TCGA-A8-A093-01 | LumA | 0 | 0 | 0.78201189 | 0 | 0.21798811 |
| TCGA-AN-A0FJ-01 | Basal | 0.88658662 | 0 | 0 | 0 | 0.11341338 |
| TCGA-A8-A07R-01 | Basal | 0.87123403 | 0.12876597 | 0 | 0 | 0 |
| TCGA-A8-A085-01 | LumB | 0 | 0.26446652 | 0.24729253 | 0.48824095 | 0 |
| TCGA-AN-A0AR-01 | Basal | 0.95988295 | 0.04011705 | 0 | 0 | 0 |
| TCGA-A8-A08F-01 | LumB | 0 | 0.22362844 | 0 | 0.77637156 | 0 |
| TCGA-A8-A07O-01 | Basal | 0.48158268 | 0.28642741 | 0 | 0.23198991 | 0 |
| TCGA-AN-A0AK-01 | LumB | 0 | 0.44849052 | 0 | 0.55150948 | 0 |
| TCGA-AN-A0FF-01 | LumB | 0 | 0 | 0.45552649 | 0.54447351 | 0 |
| TCGA-A8-A099-01 | LumA | 0 | 0 | 0.74792594 | 0.05507382 | 0.19700023 |
| TCGA-AO-A03R-01 | LumB | 0.25365965 | 0 | 0 | 0.74634035 | 0 |
| TCGA-A2-A0D0-01 | Basal | 0.81032446 | 0.17265337 | 0 | 0.01702216 | 0 |
| TCGA-AN-A0FE-01 | LumB | 0 | 0.24144737 | 0 | 0.75855263 | 0 |
| TCGA-A8-A096-01 | LumA | 0 | 0.12871535 | 0.54696202 | 0.32432263 | 0 |
| TCGA-AN-A041-01 | LumA | 0 | 0.02651515 | 0.68357078 | 0.21477838 | 0.07513569 |
| TCGA-A7-A0CD-01 | LumA | 0 | 0 | 0.7630845 | 0.03352475 | 0.20339076 |
| TCGA-A8-A095-01 | LumB | 0 | 0.16082518 | 0.16053965 | 0.67863516 | 0 |
| TCGA-A8-A08H-01 | LumB | 0 | 0 | 0.02433425 | 0.97566575 | 0 |
| TCGA-B6-A0IJ-01 | Basal | 0.68174688 | 0.1807743 | 0 | 0.13747882 | 0 |
| TCGA-AN-A0AS-01 | LumA | 0 | 0 | 0.74753528 | 0.02738579 | 0.22507894 |
| TCGA-A2-A0EY-01 | LumA | 0 | 0.30973976 | 0.36155077 | 0.32870947 | 0 |
| TCGA-BH-A0HO-01 | LumA | 0 | 0 | 0.75008807 | 0.04202104 | 0.2078909 |
| TCGA-A8-A06Z-01 | LumA | 0 | 0 | 0.52696811 | 0.47303189 | 0 |
| TCGA-AN-A04C-01 | Her2 | 0 | 0.62531138 | 0 | 0.37468862 | 0 |
| TCGA-A8-A06O-01 | LumB | 0 | 0.11167111 | 0.09424725 | 0.79408165 | 0 |
| TCGA-A8-A08Z-01 | LumA | 0 | 0 | 0.76238098 | 0.02748668 | 0.21013234 |
| TCGA-BH-A0BD-01 | LumB | 0 | 0.26068746 | 0 | 0.73931254 | 0 |
| TCGA-A8-A07C-01 | Basal | 0.82800562 | 0.0367337 | 0 | 0.13526068 | 0 |
| TCGA-A8-A079-01 | LumB | 0 | 0.14672285 | 0 | 0.85327715 | 0 |
| TCGA-A2-A04Y-01 | LumA | 0 | 0.14415835 | 0.52379284 | 0.33204881 | 0 |
| TCGA-AO-A0J4-01 | Basal | 1 | 0 | 0 | 0 | 0 |
| TCGA-AN-A03X-01 | LumA | 0 | 0 | 0.75249619 | 0.08474743 | 0.16275638 |
| TCGA-A8-A07J-01 | LumA | 0 | 0 | 0.70878658 | 0 | 0.29121342 |
| TCGA-AO-A0J3-01 | LumB | 0 | 0.07714925 | 0.3360179 | 0.58683285 | 0 |
| TCGA-A8-A07U-01 | Basal | 0.46403515 | 0.35558324 | 0 | 0.18038161 | 0 |
| TCGA-AN-A0AT-01 | Basal | 0.83777576 | 0.13291025 | 0 | 0.02931399 | 0 |
| TCGA-B6-A0IP-01 | LumA | 0 | 0 | 0.75113816 | 0 | 0.24886184 |
| TCGA-A8-A06Q-01 | LumB | 0 | 0.12878454 | 0.23982699 | 0.63138847 | 0 |
| TCGA-A2-A0D1-01 | Her2 | 0 | 0.6780412 | 0.11916051 | 0.20279829 | 0 |
| TCGA-AN-A0AJ-01 | LumB | 0 | 0.40247591 | 0 | 0.59752409 | 0 |
| TCGA-A8-A08J-01 | Her2 | 0 | 0.52617741 | 0.14544029 | 0.32838231 | 0 |
| TCGA-A8-A06N-01 | LumA | 0 | 0 | 0.67104944 | 0.02983844 | 0.29911213 |
| TCGA-A8-A0A1-01 | LumA | 0 | 0 | 0.73511643 | 0.21614171 | 0.04874186 |
| TCGA-A8-A09D-01 | LumA | 0 | 0.21813074 | 0.4683775 | 0.31349176 | 0 |
| TCGA-A8-A086-01 | LumA | 0 | 0 | 0.69371958 | 0.27795068 | 0.02832974 |
| TCGA-A8-A09G-01 | Her2 | 0 | 0.53698671 | 0.13682858 | 0.32618471 | 0 |
| TCGA-AN-A0FX-01 | Basal | 0.71728817 | 0 | 0 | 0 | 0.28271183 |
| TCGA-AO-A03P-01 | LumA | 0 | 0 | 0.61218134 | 0.38781866 | 0 |
| TCGA-A2-A0EV-01 | LumA | 0 | 0 | 0.8133642 | 0.10077819 | 0.08585761 |
| TCGA-BH-A0EE-01 | Her2 | 0 | 0.54884037 | 0 | 0.45115963 | 0 |
| TCGA-A8-A090-01 | LumA | 0 | 0 | 0.5980815 | 0.4019185 | 0 |
| TCGA-A8-A076-01 | Her2 | 0 | 0.51352361 | 0.07142524 | 0.41505115 | 0 |
| TCGA-A2-A0EO-01 | LumA | 0 | 0 | 0.63252636 | 0 | 0.36747364 |
| TCGA-B6-A0IN-01 | LumA | 0 | 0.01163107 | 0.76050877 | 0.19718406 | 0.0306761 |
| TCGA-B6-A0IM-01 | LumA | 0 | 0.04627503 | 0.60196915 | 0.35175583 | 0 |
| TCGA-B6-A0IB-01 | LumB | 0 | 0 | 0.48644435 | 0.51355565 | 0 |
| TCGA-A8-A09C-01 | LumA | 0 | 0 | 0.6283614 | 0.3716386 | 0 |
| TCGA-AN-A0FT-01 | LumA | 0 | 0.04752741 | 0.74976674 | 0.03808024 | 0.16462561 |
| TCGA-A8-A07E-01 | LumA | 0 | 0 | 0.59742729 | 0.40257271 | 0 |
| TCGA-A8-A07P-01 | LumA | 0 | 0 | 0.69466748 | 0.22276981 | 0.08256271 |
| TCGA-A8-A08A-01 | LumA | 0 | 0 | 0.79602897 | 0.0124649 | 0.19150613 |
| TCGA-A2-A04V-01 | LumA | 0 | 0 | 0.7800263 | 0.2199737 | 0 |
| TCGA-AN-A03Y-01 | LumB | 0 | 0 | 0.28868569 | 0.71131431 | 0 |
| TCGA-B6-A0IQ-01 | Basal | 1 | 0 | 0 | 0 | 0 |
| TCGA-A2-A04X-01 | Her2 | 0 | 0.58050553 | 0.27483568 | 0.14465879 | 0 |
| TCGA-A8-A09R-01 | LumB | 0 | 0.24644707 | 0.26232235 | 0.49123057 | 0 |
| TCGA-B6-A0I8-01 | LumA | 0 | 0 | 0.71849662 | 0.05908704 | 0.22241634 |
| TCGA-A2-A0CZ-01 | LumA | 0 | 0 | 0.58426016 | 0 | 0.41573985 |
| TCGA-A8-A09N-01 | LumB | 0 | 0.24018573 | 0.21722246 | 0.54259181 | 0 |
| TCGA-A8-A08P-01 | LumB | 0 | 0.28921251 | 0.14817216 | 0.56261533 | 0 |
| TCGA-A8-A08L-01 | Her2 | 0 | 0.56917023 | 0 | 0.43082977 | 0 |
| TCGA-B6-A0I9-01 | Her2 | 0 | 0.51750845 | 0 | 0.48249155 | 0 |
| TCGA-A8-A091-01 | LumA | 0 | 0 | 0.73011547 | 0 | 0.26988453 |
| TCGA-A7-A0CH-01 | LumA | 0 | 0 | 0.70471494 | 0 | 0.29528506 |
| TCGA-AN-A0FZ-01 | LumA | 0 | 0 | 0.76010672 | 0.16779769 | 0.07209559 |
| TCGA-AN-A0FK-01 | LumA | 0 | 0 | 0.62537074 | 0.37462926 | 0 |
| TCGA-A8-A094-01 | Her2 | 0 | 0.67999367 | 0.02446362 | 0.29554271 | 0 |
| TCGA-A8-A07B-01 | Her2 | 0 | 0.39051342 | 0.33157934 | 0.27790724 | 0 |
| TCGA-A2-A0CU-01 | LumA | 0 | 0 | 0.68129414 | 0.31870587 | 0 |
| TCGA-A8-A07L-01 | LumB | 0 | 0.2579982 | 0 | 0.74200181 | 0 |
| TCGA-A2-A0CY-01 | LumA | 0 | 0.09348992 | 0.57841687 | 0.08183836 | 0.24625486 |
| TCGA-AQ-A04J-01 | Basal | 0.82917654 | 0 | 0 | 0 | 0.17082346 |
| TCGA-A8-A0A2-01 | LumA | 0 | 0 | 0.70428065 | 0 | 0.29571935 |
| TCGA-AN-A0AM-01 | LumB | 0 | 0.48209664 | 0 | 0.51790337 | 0 |
| TCGA-A8-A09Q-01 | LumB | 0 | 0.08859026 | 0.42925618 | 0.48215356 | 0 |
| TCGA-A2-A0CP-01 | LumA | 0 | 0 | 0.61073478 | 0 | 0.38926522 |
| TCGA-A8-A09E-01 | LumB | 0 | 0.12116124 | 0.39500623 | 0.48383253 | 0 |
| TCGA-A7-A0CJ-01 | LumB | 0 | 0 | 0.39124477 | 0.60875523 | 0 |
| TCGA-BH-A0E6-01 | Basal | 0.81459814 | 0 | 0 | 0 | 0.18540186 |
| TCGA-A8-A09K-01 | LumA | 0 | 0 | 0.74180999 | 0.25819001 | 0 |
| TCGA-A8-A08C-01 | LumA | 0 | 0 | 0.73414859 | 0 | 0.26585141 |
| TCGA-A7-A0DB-01 | LumA | 0 | 0 | 0.72447816 | 0 | 0.27552184 |
| TCGA-A8-A07I-01 | Her2 | 0 | 0.46683463 | 0.11194127 | 0.4212241 | 0 |
| TCGA-AN-A0G0-01 | Basal | 0.64624152 | 0.17286296 | 0 | 0.18089552 | 0 |
| TCGA-AN-A0FW-01 | LumA | 0 | 0 | 0.56064759 | 0.43935241 | 0 |
| TCGA-A2-A0ET-01 | LumA | 0 | 0 | 0.64171952 | 0.1081043 | 0.25017618 |
| TCGA-AO-A03T-01 | LumA | 0 | 0 | 0.81365676 | 0 | 0.18634324 |
| TCGA-B6-A0IO-01 | LumA | 0 | 0 | 0.77264713 | 0.15825707 | 0.06909579 |
| TCGA-BH-A0HW-01 | LumA | 0 | 0 | 0.70391429 | 0.29608571 | 0 |
| TCGA-A8-A06R-01 | LumB | 0 | 0.22826377 | 0.36744683 | 0.4042894 | 0 |
| TCGA-AN-A046-01 | LumA | 0 | 0 | 0.8099966 | 0 | 0.1900034 |
| TCGA-A2-A0EX-01 | LumA | 0 | 0 | 0.67594359 | 0 | 0.32405641 |
| TCGA-A2-A0EM-01 | LumA | 0 | 0 | 0.73887677 | 0 | 0.26112323 |
| TCGA-A8-A09B-01 | LumA | 0 | 0 | 0.7603179 | 0 | 0.2396821 |
| TCGA-A2-A0D4-01 | LumB | 0 | 0.23711635 | 0 | 0.76288365 | 0 |
| TCGA-BH-A0HU-01 | LumB | 0 | 0.27075188 | 0 | 0.72924812 | 0 |
| TCGA-A8-A09W-01 | LumB | 0 | 0.38626116 | 0 | 0.61373884 | 0 |
| TCGA-BH-A18L-11 | Normal | 0 | 0 | 0.47610554 | 0 | 0.52389446 |
| TCGA-BH-A0DD-11 | LumA | 0 | 0 | 0.54453921 | 0 | 0.45546079 |
| TCGA-BH-A1EU-11 | Normal | 0 | 0 | 0.48828722 | 0 | 0.51171278 |
| TCGA-AR-A1AR-01 | Basal | 0.6535163 | 0.13067785 | 0 | 0.21580586 | 0 |
| TCGA-AR-A1AS-01 | LumA | 0 | 0.06708899 | 0.75156465 | 0.13501756 | 0.04632881 |
| TCGA-BH-A0B5-11 | LumA | 0 | 0 | 0.52278481 | 0 | 0.47721519 |
| TCGA-BH-A18J-01 | LumA | 0 | 0 | 0.59958814 | 0.40041186 | 0 |
| TCGA-E2-A15K-06 | LumA | 0 | 0 | 0.67817444 | 0.21849086 | 0.10333471 |
| TCGA-E2-A14W-01 | LumA | 0 | 0.18252015 | 0.43249894 | 0.38498091 | 0 |
| TCGA-BH-A18R-11 | LumA | 0 | 0 | 0.5037339 | 0 | 0.4962661 |
| TCGA-E2-A1B1-01 | LumA | 0 | 0.11801058 | 0.56038958 | 0 | 0.32159984 |
| TCGA-AR-A1AH-01 | Basal | 1 | 0 | 0 | 0 | 0 |
| TCGA-E2-A14S-01 | LumB | 0 | 0 | 0.47822843 | 0.52177157 | 0 |
| TCGA-BH-A1ES-01 | LumA | 0 | 0.06737752 | 0.81450501 | 0.11811747 | 0 |
| TCGA-BH-A0BF-01 | LumB | 0 | 0.29958894 | 0.15321996 | 0.5471911 | 0 |
| TCGA-BH-A0HA-11 | Normal | 0 | 0 | 0.45929995 | 0 | 0.54070006 |
| TCGA-BH-A18M-11 | LumA | 0 | 0 | 0.50154365 | 0 | 0.49845635 |
| TCGA-E2-A1AZ-01 | Basal | 0.62829125 | 0.31650066 | 0 | 0.05520809 | 0 |
| TCGA-BH-A18H-01 | LumA | 0 | 0 | 0.69702944 | 0 | 0.30297056 |
| TCGA-AR-A1AX-01 | LumA | 0 | 0.16788431 | 0.63711682 | 0.19499887 | 0 |
| TCGA-BH-A18N-11 | LumA | 0 | 0 | 0.51728676 | 0 | 0.48271324 |
| TCGA-E2-A150-01 | Basal | 0.77264169 | 0 | 0 | 0 | 0.22735831 |
| TCGA-BH-A18S-01 | LumA | 0 | 0 | 0.71023382 | 0 | 0.28976618 |
| TCGA-BH-A1EV-01 | Her2 | 0 | 0.39494037 | 0.28686193 | 0.3181977 | 0 |
| TCGA-BH-A18N-01 | LumA | 0 | 0 | 0.74120792 | 0 | 0.25879209 |
| TCGA-AR-A1AN-01 | LumA | 0 | 0 | 0.57436138 | 0 | 0.42563862 |
| TCGA-E2-A15L-01 | LumA | 0 | 0 | 0.60231139 | 0.39768861 | 0 |
| TCGA-E2-A153-01 | LumA | 0 | 0 | 0.67092719 | 0 | 0.32907281 |
| TCGA-E2-A15M-01 | LumB | 0 | 0.03074276 | 0.29972324 | 0.669534 | 0 |
| TCGA-BH-A1F0-01 | Basal | 0.926097 | 0.06089705 | 0 | 0.01300596 | 0 |
| TCGA-AR-A1AI-01 | Basal | 0.95304878 | 0.04695122 | 0 | 0 | 0 |
| TCGA-E2-A1B4-01 | LumA | 0 | 0 | 0.70391957 | 0 | 0.29608043 |
| TCGA-E2-A14Q-01 | LumA | 0 | 0 | 0.680442 | 0 | 0.319558 |
| TCGA-BH-A0DT-01 | LumA | 0 | 0 | 0.66447153 | 0 | 0.33552847 |
| TCGA-E2-A14N-01 | Basal | 0.929198 | 0.07080201 | 0 | 0 | 0 |
| TCGA-BH-A18L-01 | LumB | 0 | 0.10638462 | 0 | 0.89361539 | 0 |
| TCGA-C8-A1HF-01 | Her2 | 0 | 0.62529713 | 0 | 0.37470287 | 0 |
| TCGA-E2-A15E-06 | LumA | 0 | 0 | 0.70519631 | 0 | 0.29480369 |
| TCGA-E2-A1B0-01 | Her2 | 0.01738441 | 0.82327058 | 0 | 0.15934502 | 0 |
| TCGA-AR-A1AQ-01 | Basal | 0.9985642 | 0.0014358 | 0 | 0 | 0 |
| TCGA-BH-A18U-01 | LumB | 0 | 0.43167253 | 0 | 0.56832747 | 0 |
| TCGA-BH-A0DI-01 | LumA | 0 | 0 | 0.5959632 | 0 | 0.4040368 |
| TCGA-BH-A0BS-01 | LumA | 0 | 0 | 0.66753623 | 0 | 0.33246377 |
| TCGA-BH-A18U-11 | Normal | 0 | 0 | 0.48310764 | 0 | 0.51689236 |
| TCGA-BH-A0BZ-11 | LumA | 0 | 0 | 0.52160516 | 0 | 0.47839484 |
| TCGA-AR-A1AU-01 | LumA | 0 | 0 | 0.63053052 | 0 | 0.36946948 |
| TCGA-BH-A0AU-01 | LumB | 0 | 0.19131387 | 0.27920613 | 0.52947999 | 0 |
| TCGA-E2-A15I-11 | Normal | 0 | 0 | 0.47469416 | 0 | 0.52530584 |
| TCGA-E2-A15H-01 | LumA | 0 | 0.0983483 | 0.6094853 | 0.2921664 | 0 |
| TCGA-E2-A1B5-01 | Basal | 0.697453 | 0 | 0 | 0 | 0.302547 |
| TCGA-BH-A1ET-11 | Normal | 0 | 0 | 0.49837258 | 0 | 0.50162742 |
| TCGA-E2-A153-11 | Normal | 0 | 0.01965994 | 0 | 0 | 0.98034006 |
| TCGA-BH-A0C3-11 | Normal | 0 | 0 | 0.44100285 | 0 | 0.55899715 |
| TCGA-BH-A1F0-11 | Normal | 0 | 0 | 0.48848936 | 0 | 0.51151064 |
| TCGA-E2-A14V-01 | Her2 | 0 | 0.55940985 | 0 | 0.44059015 | 0 |
| TCGA-E2-A1BC-01 | LumA | 0 | 0 | 0.67836372 | 0 | 0.32163628 |
| TCGA-E2-A15K-01 | LumB | 0 | 0.28189122 | 0 | 0.71810878 | 0 |
| TCGA-C8-A1HG-01 | LumB | 0 | 0 | 0 | 1 | 0 |
| TCGA-E2-A15G-01 | LumA | 0 | 0 | 0.68674652 | 0 | 0.31325348 |
| TCGA-C8-A12Y-01 | LumA | 0 | 0 | 0.55742543 | 0.44257457 | 0 |
| TCGA-E2-A1BD-01 | LumA | 0 | 0 | 0.74072028 | 0.02687227 | 0.23240745 |
| TCGA-E2-A15J-01 | LumA | 0 | 0 | 0.74190652 | 0.04384696 | 0.21424652 |
| TCGA-E2-A158-01 | Normal | 0.30560939 | 0 | 0.18345423 | 0 | 0.51093638 |
| TCGA-BH-A0HA-01 | LumA | 0 | 0 | 0.63893389 | 0 | 0.36106611 |
| TCGA-E2-A14P-01 | Her2 | 0 | 0.72338392 | 0 | 0.27661608 | 0 |
| TCGA-BH-A0DO-01 | LumA | 0 | 0 | 0.59421842 | 0 | 0.40578159 |
| TCGA-BH-A18I-01 | LumA | 0 | 0 | 0.67383732 | 0 | 0.32616268 |
| TCGA-E2-A1B6-01 | LumA | 0 | 0 | 0.64925109 | 0 | 0.35074891 |
| TCGA-AR-A1AK-01 | LumA | 0 | 0 | 0.66638698 | 0.01974604 | 0.31386698 |
| TCGA-E2-A152-01 | LumA | 0 | 0.34457801 | 0.56611492 | 0 | 0.08930707 |
| TCGA-BH-A18F-01 | LumB | 0 | 0.28033247 | 0 | 0.71966753 | 0 |
| TCGA-A7-A13F-11 | Normal | 0 | 0 | 0.46138676 | 0 | 0.53861325 |
| TCGA-BH-A0C1-01 | LumA | 0 | 0 | 0.68480449 | 0.31519552 | 0 |
| TCGA-A7-A13F-01 | LumB | 0 | 0.44686422 | 0 | 0.55313578 | 0 |
| TCGA-E2-A155-01 | LumB | 0 | 0.01556262 | 0.34897743 | 0.63545995 | 0 |
| TCGA-E2-A14Y-01 | Basal | 0.76805361 | 0.23194639 | 0 | 0 | 0 |
| TCGA-A7-A13E-01 | Basal | 0.76632954 | 0 | 0 | 0 | 0.23367046 |
| TCGA-BH-A1EW-01 | LumB | 0 | 0.09016651 | 0.3815897 | 0.5282438 | 0 |
| TCGA-E2-A15A-06 | LumB | 0 | 0.35011458 | 0 | 0.64988542 | 0 |
| TCGA-BH-A18K-11 | Normal | 0 | 0 | 0.47113093 | 0 | 0.52886907 |
| TCGA-E2-A15E-01 | LumA | 0 | 0 | 0.58556542 | 0 | 0.41443458 |
| TCGA-AR-A1AW-01 | LumA | 0 | 0 | 0.72978882 | 0.12506832 | 0.14514286 |
| TCGA-BH-A0C3-01 | LumB | 0 | 0.12333779 | 0.29782593 | 0.57883628 | 0 |
| TCGA-BH-A0B5-01 | LumB | 0 | 0.28074753 | 0 | 0.71925247 | 0 |
| TCGA-BH-A0DO-11 | Normal | 0 | 0 | 0.44722241 | 0 | 0.55277759 |
| TCGA-BH-A18K-01 | Basal | 0.83040728 | 0.13185017 | 0 | 0.03774256 | 0 |
| TCGA-A7-A13D-01 | Basal | 0.83738665 | 0.16261336 | 0 | 0 | 0 |
| TCGA-AR-A1AV-01 | LumB | 0 | 0.08172559 | 0.41127764 | 0.50699677 | 0 |
| TCGA-C8-A133-01 | LumA | 0 | 0 | 0.64103259 | 0 | 0.35896741 |
| TCGA-BH-A18T-01 | LumA | 0 | 0 | 0.68015013 | 0.31984988 | 0 |
| TCGA-E2-A15I-01 | LumA | 0 | 0 | 0.67186523 | 0 | 0.32813477 |
| TCGA-BH-A0BT-01 | LumA | 0 | 0 | 0.74233766 | 0.06096812 | 0.19669422 |
| TCGA-C8-A1HI-01 | LumA | 0 | 0 | 0.63619993 | 0 | 0.36380007 |
| TCGA-AR-A1AJ-01 | Basal | 0.63816279 | 0.15128982 | 0 | 0.21054739 | 0 |
| TCGA-BH-A18R-01 | Her2 | 0 | 0.59968217 | 0 | 0.40031783 | 0 |
| TCGA-AR-A1AY-01 | Basal | 1 | 0 | 0 | 0 | 0 |
| TCGA-BH-A0DD-01 | LumB | 0 | 0.29730492 | 0 | 0.70269508 | 0 |
| TCGA-BH-A18Q-01 | Basal | 0.96974712 | 0 | 0 | 0 | 0.03025288 |
| TCGA-BH-A0H3-01 | LumA | 0 | 0 | 0.65249799 | 0 | 0.34750202 |
| TCGA-AR-A1AO-01 | Normal | 0 | 0 | 0.39131604 | 0 | 0.60868396 |
| TCGA-BH-A1EW-11 | LumA | 0 | 0 | 0.60679368 | 0 | 0.39320632 |
| TCGA-BH-A0BO-01 | LumA | 0 | 0 | 0.56229804 | 0 | 0.43770196 |
| TCGA-BH-A1EO-11 | LumA | 0 | 0 | 0.55114929 | 0 | 0.44885071 |
| TCGA-BH-A18J-11 | Normal | 0 | 0 | 0.47454225 | 0 | 0.52545775 |
| TCGA-BH-A0AZ-01 | LumA | 0 | 0 | 0.6537957 | 0 | 0.3462043 |
| TCGA-BH-A0DV-11 | Normal | 0 | 0 | 0.43107711 | 0 | 0.56892289 |
| TCGA-BH-A0DT-11 | LumA | 0 | 0 | 0.50790981 | 0 | 0.49209019 |
| TCGA-E2-A156-01 | LumA | 0 | 0 | 0.72946176 | 0 | 0.27053824 |
| TCGA-AR-A1AT-01 | Her2 | 0 | 0.58755552 | 0 | 0.41244449 | 0 |
| TCGA-BH-A18P-11 | Normal | 0 | 0 | 0.47897247 | 0 | 0.52102753 |
| TCGA-BH-A18V-11 | Normal | 0 | 0 | 0.44383039 | 0 | 0.55616961 |
| TCGA-E2-A158-11 | LumA | 0 | 0 | 0.50233057 | 0 | 0.49766943 |
| TCGA-BH-A18G-01 | Basal | 0.81138641 | 0 | 0 | 0 | 0.18861359 |
| TCGA-E2-A15A-01 | LumB | 0 | 0.32245502 | 0 | 0.67754498 | 0 |
| TCGA-AR-A1AP-01 | LumA | 0 | 0 | 0.81369416 | 0.13646563 | 0.04984021 |
| TCGA-BH-A1ET-01 | LumA | 0 | 0 | 0.69723708 | 0 | 0.30276292 |
| TCGA-E2-A1BC-11 | LumA | 0 | 0 | 0.53375261 | 0 | 0.4662474 |
| TCGA-BH-A18P-01 | Her2 | 0 | 0.4778104 | 0.05439475 | 0.46779485 | 0 |
| TCGA-BH-A18M-01 | LumA | 0 | 0 | 0.73084989 | 0 | 0.26915011 |
| TCGA-AR-A1AL-01 | LumA | 0 | 0 | 0.71945936 | 0 | 0.28054064 |
| TCGA-BH-A18Q-11 | Normal | 0 | 0 | 0.4527027 | 0 | 0.5472973 |
| TCGA-BH-A0DG-01 | LumA | 0 | 0.04412845 | 0.63188464 | 0.32398692 | 0 |
| TCGA-C8-A1HM-01 | LumB | 0 | 0.40941042 | 0 | 0.59058958 | 0 |
| TCGA-BH-A1EO-01 | LumA | 0 | 0.01913965 | 0.78759352 | 0.13435162 | 0.05891521 |
| TCGA-C8-A1HN-01 | LumB | 0 | 0.2627968 | 0 | 0.7372032 | 0 |
| TCGA-BH-A0BZ-01 | Her2 | 0 | 0.51772449 | 0 | 0.48227551 | 0 |
| TCGA-BH-A18S-11 | Normal | 0 | 0 | 0.46105256 | 0 | 0.53894744 |
| TCGA-BH-A18V-01 | Basal | 0.85170697 | 0.14829303 | 0 | 0 | 0 |
| TCGA-E2-A15C-01 | LumA | 0 | 0 | 0.67195951 | 0 | 0.32804049 |
| TCGA-E2-A15M-11 | Normal | 0 | 0 | 0.48879742 | 0 | 0.51120258 |
| TCGA-C8-A1HL-01 | LumB | 0 | 0.4687885 | 0 | 0.5312115 | 0 |
| TCGA-BH-A1EU-01 | LumA | 0 | 0 | 0.65307352 | 0 | 0.34692648 |
| TCGA-A7-A13E-11 | LumA | 0 | 0 | 0.50309074 | 0 | 0.49690927 |
| TCGA-BH-A0AU-11 | Normal | 0 | 0 | 0.46746379 | 0 | 0.53253621 |
| TCGA-BH-A0BS-11 | Normal | 0 | 0 | 0.44932525 | 0 | 0.55067476 |
| TCGA-A2-A04W-01 | Her2 | 0 | 0.67475819 | 0.09803432 | 0.22720749 | 0 |
| TCGA-AO-A12F-01 | Basal | 0.89075096 | 0 | 0 | 0 | 0.10924905 |
| TCGA-BH-A0DL-11 | Normal | 0 | 0 | 0.45308809 | 0 | 0.54691191 |
| TCGA-A1-A0SD-01 | LumA | 0 | 0 | 0.75928466 | 0 | 0.24071534 |
| TCGA-C8-A12W-01 | LumB | 0 | 0.46351567 | 0 | 0.53648433 | 0 |
| TCGA-D8-A142-01 | Basal | 0.69119654 | 0 | 0 | 0 | 0.30880346 |
| TCGA-C8-A12X-01 | LumA | 0 | 0 | 0.81537561 | 0.18462439 | 0 |
| TCGA-A2-A0CS-01 | LumA | 0 | 0 | 0.78392808 | 0 | 0.21607192 |
| TCGA-BH-A0E9-01 | LumA | 0 | 0 | 0.56147223 | 0 | 0.43852777 |
| TCGA-B6-A0WS-01 | LumA | 0 | 0 | 0.74868884 | 0.0262233 | 0.22508786 |
| TCGA-A7-A0DA-01 | Basal | 0.5583085 | 0 | 0 | 0 | 0.44169151 |
| TCGA-E2-A14O-01 | LumA | 0 | 0.10614968 | 0.53722175 | 0.35662858 | 0 |
| TCGA-E2-A14X-01 | Basal | 0.79943725 | 0 | 0 | 0 | 0.20056275 |
| TCGA-D8-A13Y-01 | LumB | 0.24077625 | 0.25857176 | 0 | 0.50065199 | 0 |
| TCGA-C8-A138-01 | Her2 | 0 | 0.49001127 | 0.40600348 | 0.10398525 | 0 |
| TCGA-E2-A15D-01 | LumA | 0 | 0 | 0.6755849 | 0 | 0.3244151 |
| TCGA-A2-A0ES-01 | LumA | 0 | 0 | 0.53844763 | 0 | 0.46155237 |
| TCGA-BH-A0BP-01 | LumA | 0 | 0 | 0.62411593 | 0 | 0.37588407 |
| TCGA-AR-A0TS-01 | Basal | 1 | 0 | 0 | 0 | 0 |
| TCGA-A2-A0EW-01 | LumA | 0 | 0 | 0.61291391 | 0 | 0.38708609 |
| TCGA-D8-A143-01 | Basal | 0.4677304 | 0.38244842 | 0 | 0.14982118 | 0 |
| TCGA-D8-A145-01 | LumA | 0 | 0 | 0.59151403 | 0 | 0.40848597 |
| TCGA-A2-A0D3-01 | LumA | 0 | 0 | 0.69264729 | 0 | 0.30735271 |
| TCGA-A2-A0T3-01 | LumB | 0 | 0.24955874 | 0 | 0.75044126 | 0 |
| TCGA-BH-A0AV-01 | Basal | 1 | 0 | 0 | 0 | 0 |
| TCGA-BH-A0BW-11 | Normal | 0 | 0 | 0.35678643 | 0 | 0.64321357 |
| TCGA-BH-A0B0-01 | LumA | 0 | 0 | 0.66334098 | 0 | 0.33665902 |
| TCGA-E2-A154-01 | LumA | 0 | 0 | 0.7434837 | 0.19105404 | 0.06546226 |
| TCGA-E2-A159-01 | Her2 | 0.37148292 | 0.49274788 | 0 | 0.1357692 | 0 |
| TCGA-E2-A15F-01 | LumA | 0 | 0 | 0.75625915 | 0.05384793 | 0.18989292 |
| TCGA-D8-A140-01 | LumA | 0 | 0 | 0.63676599 | 0.31614431 | 0.0470897 |
| TCGA-A2-A0CV-01 | LumA | 0 | 0 | 0.62052011 | 0 | 0.3794799 |
| TCGA-AO-A12A-01 | LumA | 0 | 0 | 0.62519219 | 0 | 0.37480781 |
| TCGA-D8-A146-01 | LumA | 0 | 0 | 0.66852997 | 0 | 0.33147003 |
| TCGA-A2-A0CW-01 | LumB | 0 | 0.34830823 | 0 | 0.65169177 | 0 |
| TCGA-A2-A04N-01 | LumA | 0 | 0 | 0.80117872 | 0 | 0.19882128 |
| TCGA-E2-A15S-01 | LumB | 0 | 0.19621266 | 0 | 0.80378734 | 0 |
| TCGA-D8-A147-01 | Basal | 0.97036531 | 0.02963469 | 0 | 0 | 0 |
| TCGA-E2-A10A-01 | LumB | 0 | 0.0353675 | 0.46954628 | 0.49508621 | 0 |
| TCGA-BH-A0EI-01 | LumA | 0 | 0 | 0.61434457 | 0 | 0.38565543 |
| TCGA-BH-A0DE-01 | LumA | 0 | 0 | 0.65877875 | 0 | 0.34122125 |
| TCGA-C8-A137-01 | Her2 | 0 | 0.68862826 | 0 | 0.31137174 | 0 |
| TCGA-B6-A0RH-01 | LumA | 0 | 0.42555735 | 0.43076166 | 0.14368099 | 0 |
| TCGA-BH-A0BW-01 | Basal | 0.64070985 | 0.35929015 | 0 | 0 | 0 |
| TCGA-BH-A0BG-01 | Basal | 0.68355255 | 0 | 0 | 0 | 0.31644745 |
| TCGA-C8-A12O-01 | LumA | 0 | 0.33635206 | 0.44898826 | 0.21465969 | 0 |
| TCGA-BH-A0H5-11 | Normal | 0 | 0 | 0.45459682 | 0 | 0.54540318 |
| TCGA-BH-A0DL-01 | Basal | 0.92216057 | 0.07783943 | 0 | 0 | 0 |
| TCGA-BH-A0DX-01 | LumA | 0 | 0 | 0.76031208 | 0.15354608 | 0.08614184 |
| TCGA-B6-A0RQ-01 | Normal | 0 | 0 | 0.49814827 | 0 | 0.50185173 |
| TCGA-E2-A15P-01 | LumA | 0 | 0 | 0.66129155 | 0 | 0.33870845 |
| TCGA-BH-A0H5-01 | LumA | 0 | 0 | 0.67825975 | 0 | 0.32174025 |
| TCGA-AO-A03V-01 | LumA | 0 | 0 | 0.68194784 | 0 | 0.31805216 |
| TCGA-BH-A0EA-01 | LumA | 0 | 0 | 0.59929026 | 0 | 0.40070974 |
| TCGA-C8-A130-01 | LumB | 0 | 0.49302139 | 0 | 0.50697861 | 0 |
| TCGA-E2-A15T-01 | LumB | 0 | 0 | 0.37576578 | 0.62423422 | 0 |
| TCGA-E2-A15O-01 | LumB | 0 | 0 | 0.45527523 | 0.54472477 | 0 |
| TCGA-BH-A0B7-01 | Her2 | 0 | 0.49189996 | 0.46353117 | 0 | 0.04456887 |
| TCGA-AO-A12D-01 | Her2 | 0 | 0.59559719 | 0.11679103 | 0.28761179 | 0 |
| TCGA-A2-A0CL-01 | Her2 | 0 | 0.56938584 | 0 | 0.43061416 | 0 |
| TCGA-BH-A0C7-01 | LumB | 0 | 0.2126935 | 0 | 0.7873065 | 0 |
| TCGA-C8-A12U-01 | LumB | 0 | 0.30393511 | 0 | 0.6960649 | 0 |
| TCGA-A2-A04U-01 | Basal | 1 | 0 | 0 | 0 | 0 |
| TCGA-C8-A12V-01 | Basal | 1 | 0 | 0 | 0 | 0 |
| TCGA-BH-A0BQ-11 | Normal | 0 | 0 | 0.43521916 | 0 | 0.56478084 |
| TCGA-C8-A12L-01 | Her2 | 0.1533647 | 0.49341736 | 0 | 0.35321794 | 0 |
| TCGA-C8-A134-01 | Basal | 0.9590686 | 0.04093141 | 0 | 0 | 0 |
| TCGA-BH-A0B7-11 | Normal | 0 | 0 | 0.44453046 | 0 | 0.55546954 |
| TCGA-BH-A0W7-01 | LumA | 0 | 0.1517515 | 0.51885968 | 0.32938883 | 0 |
| TCGA-D8-A13Z-01 | Her2 | 0.19420975 | 0.5977548 | 0 | 0.20803545 | 0 |
| TCGA-C8-A12N-01 | LumA | 0 | 0 | 0.70187756 | 0 | 0.29812244 |
| TCGA-C8-A12P-01 | Her2 | 0 | 0.65253934 | 0 | 0.34746066 | 0 |
| TCGA-BH-A0BR-01 | LumA | 0 | 0.18911677 | 0.71452128 | 0.09636195 | 0 |
| TCGA-E2-A14Z-01 | LumA | 0 | 0 | 1 | 0 | 0 |
| TCGA-E2-A15R-01 | LumA | 0 | 0 | 0.61121363 | 0.38878637 | 0 |
| TCGA-AR-A0TY-01 | LumB | 0 | 0.36010902 | 0 | 0.63989098 | 0 |
| TCGA-BH-A0BQ-01 | LumA | 0 | 0 | 0.6225956 | 0 | 0.3774044 |
| TCGA-C8-A132-01 | LumA | 0 | 0 | 0.71090798 | 0 | 0.28909202 |
| TCGA-BH-A0BL-01 | Basal | 0.96771307 | 0 | 0 | 0 | 0.03228693 |
| TCGA-C8-A12Q-01 | Her2 | 0 | 0.49949963 | 0.40182801 | 0.09867236 | 0 |
| TCGA-B6-A0X0-01 | LumA | 0 | 0 | 0.66577625 | 0 | 0.33422375 |
| TCGA-C8-A12T-01 | Her2 | 0 | 0.54730447 | 0 | 0.45269553 | 0 |
| TCGA-C8-A12K-01 | Basal | 0.83276074 | 0.03558696 | 0 | 0.1316523 | 0 |
| TCGA-C8-A131-01 | Basal | 0.43776534 | 0.40786316 | 0 | 0.1543715 | 0 |
| TCGA-C8-A12M-01 | LumA | 0 | 0 | 0.72641903 | 0.27358097 | 0 |
| TCGA-E2-A14T-01 | LumA | 0 | 0 | 0.84299497 | 0.04477612 | 0.11222891 |
| TCGA-C8-A135-01 | Her2 | 0 | 0.73392476 | 0 | 0.26607524 | 0 |
| TCGA-B6-A0IH-01 | LumA | 0 | 0 | 0.61813937 | 0 | 0.38186063 |
| TCGA-AR-A0U1-01 | Basal | 0.80713807 | 0 | 0 | 0 | 0.19286193 |
| TCGA-D8-A141-01 | LumA | 0 | 0 | 0.73489562 | 0 | 0.26510438 |
| TCGA-C8-A12Z-01 | Her2 | 0 | 0.62731253 | 0 | 0.37268747 | 0 |
| TCGA-E2-A14R-01 | Basal | 0.73612677 | 0.1378373 | 0 | 0.12603593 | 0 |
| TCGA-AO-A12H-01 | LumA | 0 | 0 | 0.7220575 | 0 | 0.2779425 |
| TCGA-AN-A0AL-01 | Basal | 0.79503047 | 0.09723394 | 0 | 0.10773558 | 0 |
| TCGA-A8-A0A6-01 | LumA | 0 | 0 | 0.61807276 | 0 | 0.38192725 |
| TCGA-BH-A0DS-01 | LumA | 0 | 0 | 0.71254802 | 0 | 0.28745199 |
| TCGA-AO-A0JM-01 | LumB | 0 | 0.49243785 | 0 | 0.50756215 | 0 |
| TCGA-BH-A0GZ-01 | LumA | 0 | 0 | 0.79003363 | 0.17713238 | 0.032834 |
| TCGA-AO-A0JL-01 | Basal | 0.88037577 | 0.03111264 | 0 | 0 | 0.08851159 |
| TCGA-BH-A0H0-01 | LumB | 0 | 0.21713208 | 0 | 0.78286793 | 0 |
| TCGA-B6-A0IK-01 | Her2 | 0 | 0.9185656 | 0.0814344 | 0 | 0 |
| TCGA-B6-A0RE-01 | Basal | 1 | 0 | 0 | 0 | 0 |
| TCGA-A2-A0CT-01 | LumA | 0 | 0.20543111 | 0.44345865 | 0.35111024 | 0 |
| TCGA-B6-A0RI-01 | LumB | 0 | 0.04519324 | 0.45626603 | 0.49854073 | 0 |
| TCGA-BH-A0DP-01 | LumA | 0 | 0 | 0.64677457 | 0 | 0.35322543 |
| TCGA-AO-A0JD-01 | LumB | 0 | 0.14116031 | 0 | 0.85883969 | 0 |
| TCGA-BH-A0E0-01 | Basal | 0.81814576 | 0.17739217 | 0 | 0.00446207 | 0 |
| TCGA-AO-A0JC-01 | LumA | 0 | 0 | 0.70852737 | 0.00038171 | 0.29109092 |
| TCGA-BH-A0HF-01 | LumA | 0 | 0 | 0.71474303 | 0 | 0.28525697 |
| TCGA-AO-A0JA-01 | LumA | 0 | 0.05449501 | 0.52440716 | 0.42109783 | 0 |
| TCGA-BH-A0E2-01 | LumA | 0 | 0 | 0.59590092 | 0.40409908 | 0 |
| TCGA-BH-A0B9-01 | Basal | 0.96236128 | 0 | 0 | 0 | 0.03763872 |
| TCGA-AO-A0JB-01 | Normal | 0 | 0 | 0.34722641 | 0 | 0.65277359 |
| TCGA-BH-A0HY-01 | Her2 | 0 | 0.54063285 | 0.00380228 | 0.45556486 | 0 |
| TCGA-BH-A0HX-01 | LumA | 0 | 0 | 0.70169275 | 0.29338869 | 0.00491856 |
| TCGA-BH-A0B1-01 | LumA | 0 | 0 | 0.7195333 | 0.13057556 | 0.14989114 |
| TCGA-AO-A03L-01 | Her2 | 0 | 0.48546736 | 0.07083698 | 0.44369566 | 0 |
| TCGA-BH-A0E1-01 | LumB | 0 | 0 | 0.33539554 | 0.66460446 | 0 |
| TCGA-BH-A0B8-01 | LumA | 0 | 0 | 0.70738006 | 0.147033 | 0.14558694 |
| TCGA-AO-A0JJ-01 | LumA | 0 | 0 | 0.66988046 | 0 | 0.33011954 |
| TCGA-BH-A0AW-01 | Her2 | 0 | 0.59713384 | 0.09662585 | 0.30624031 | 0 |
| TCGA-BH-A0H6-01 | LumA | 0 | 0 | 0.70133685 | 0 | 0.29866315 |
| TCGA-AO-A0JF-01 | LumA | 0 | 0 | 0.75307772 | 0 | 0.24692228 |
| TCGA-A7-A0D9-01 | LumA | 0 | 0 | 0.65715797 | 0 | 0.34284203 |
| TCGA-BH-A0HB-01 | LumB | 0 | 0 | 0.47901252 | 0.52098748 | 0 |
| TCGA-AO-A0JI-01 | LumA | 0 | 0 | 0.56270114 | 0.43729886 | 0 |
| TCGA-AO-A0JE-01 | Her2 | 0 | 0.62106725 | 0.11927782 | 0.25965493 | 0 |
| TCGA-B6-A0RG-01 | LumA | 0 | 0 | 0.69412359 | 0.26970902 | 0.03616739 |
| TCGA-A8-A0AD-01 | LumA | 0 | 0 | 0.75815904 | 0 | 0.24184096 |
| TCGA-BH-A0BM-01 | LumA | 0 | 0 | 0.64265009 | 0 | 0.35734991 |
| TCGA-BH-A0GY-01 | LumA | 0 | 0 | 0.73386648 | 0 | 0.26613352 |
| TCGA-BH-A0H7-01 | LumA | 0 | 0.02106886 | 0.6804434 | 0.29415651 | 0.00433123 |
| TCGA-BH-A0BJ-01 | LumA | 0 | 0 | 0.68365071 | 0 | 0.3163493 |
| TCGA-A2-A0EU-01 | LumA | 0 | 0 | 0.75992427 | 0.17032479 | 0.06975093 |
| TCGA-A8-A08O-01 | LumA | 0 | 0 | 0.66463585 | 0 | 0.33536415 |
| TCGA-BH-A0BA-01 | LumA | 0 | 0 | 0.72953163 | 0 | 0.27046838 |
| TCGA-BH-A0DK-01 | LumA | 0 | 0 | 0.53920612 | 0.46079388 | 0 |
| TCGA-BH-A0H9-01 | LumA | 0 | 0 | 0.69756867 | 0.28903171 | 0.01339962 |
| TCGA-BH-A0HK-01 | LumA | 0 | 0 | 0.63978579 | 0 | 0.36021421 |
| TCGA-BH-A0C0-01 | LumB | 0 | 0.18982768 | 0 | 0.81017232 | 0 |
| TCGA-BH-A0B3-01 | Basal | 0.90012672 | 0.09987328 | 0 | 0 | 0 |
| TCGA-AR-A0TX-01 | Her2 | 0 | 0.57087214 | 0.1913068 | 0.23782106 | 0 |
| TCGA-BH-A0BC-11 | Normal | 0 | 0 | 0.49086338 | 0 | 0.50913662 |
| TCGA-AR-A0TW-01 | LumA | 0 | 0 | 0.79475665 | 0.10044245 | 0.1048009 |
| TCGA-A1-A0SP-01 | Basal | 1 | 0 | 0 | 0 | 0 |
| TCGA-BH-A0C0-11 | Normal | 0 | 0 | 0.46333964 | 0 | 0.53666036 |
| TCGA-AR-A0TP-01 | Basal | 0.83165042 | 0 | 0 | 0 | 0.16834958 |
| TCGA-B6-A0RP-01 | LumA | 0 | 0 | 0.70197978 | 0 | 0.29802022 |
| TCGA-A7-A0D9-11 | LumA | 0 | 0 | 0.53847644 | 0 | 0.46152356 |
| TCGA-B6-A0RM-01 | LumA | 0 | 0 | 0.78939743 | 0.15930194 | 0.05130063 |
| TCGA-AR-A0TV-01 | LumB | 0 | 0.01988256 | 0.11198105 | 0.8681364 | 0 |
| TCGA-AR-A0TR-01 | LumA | 0 | 0 | 0.65553298 | 0 | 0.34446702 |
| TCGA-BH-A0DH-11 | LumA | 0 | 0 | 0.50170952 | 0 | 0.49829048 |
| TCGA-BH-A0DZ-11 | Normal | 0 | 0 | 0.46288892 | 0 | 0.53711108 |
| TCGA-A2-A0T6-01 | LumA | 0 | 0 | 0.71864187 | 0 | 0.28135813 |
| TCGA-A2-A0SX-01 | Basal | 0.87703583 | 0 | 0 | 0 | 0.12296417 |
| TCGA-BH-A0HI-01 | LumA | 0 | 0 | 0.74378745 | 0.00450555 | 0.251707 |
| TCGA-BH-A0HK-11 | Normal | 0 | 0 | 0.47133347 | 0 | 0.52866653 |
| TCGA-A2-A0T2-01 | Normal | 0.26853737 | 0 | 0.24112242 | 0 | 0.4903402 |
| TCGA-AR-A0TT-01 | LumB | 0 | 0.08316094 | 0.42954075 | 0.4872983 | 0 |
| TCGA-AR-A0TQ-01 | Her2 | 0 | 0.52729101 | 0 | 0.47270899 | 0 |
| TCGA-A2-A0T4-01 | LumB | 0 | 0.0320327 | 0.4729097 | 0.4950576 | 0 |
| TCGA-BH-A0E0-11 | LumA | 0 | 0 | 0.50172487 | 0 | 0.49827513 |
| TCGA-BH-A0H9-11 | LumA | 0 | 0 | 0.52738392 | 0 | 0.47261608 |
| TCGA-BH-A0B8-11 | LumA | 0 | 0 | 0.50479729 | 0 | 0.49520271 |
| TCGA-AR-A0TZ-01 | LumB | 0 | 0.12284416 | 0.38559243 | 0.49156341 | 0 |
| TCGA-BH-A0E1-11 | Normal | 0 | 0 | 0.44627566 | 0 | 0.55372434 |
| TCGA-B6-A0RL-01 | LumB | 0 | 0.2363024 | 0 | 0.76369761 | 0 |
| TCGA-A7-A0DC-11 | Normal | 0 | 0 | 0.48699567 | 0 | 0.51300434 |
| TCGA-BH-A0DP-11 | Normal | 0 | 0 | 0.48704148 | 0 | 0.51295852 |
| TCGA-A1-A0SJ-01 | LumA | 0 | 0.10750271 | 0.55859557 | 0.25864207 | 0.07525965 |
| TCGA-BH-A0AY-11 | Normal | 0 | 0 | 0.47690468 | 0 | 0.52309532 |
| TCGA-B6-A0RO-01 | LumA | 0 | 0 | 0.68454125 | 0 | 0.31545875 |
| TCGA-BH-A0DQ-11 | LumA | 0 | 0 | 0.52410751 | 0 | 0.47589249 |
| TCGA-B6-A0RS-01 | Her2 | 0 | 0.71155512 | 0 | 0.28844488 | 0 |
| TCGA-BH-A0BM-11 | Normal | 0 | 0 | 0.43639648 | 0 | 0.56360352 |
| TCGA-A1-A0SO-01 | Basal | 0.34082397 | 0.33858539 | 0 | 0.32059064 | 0 |
| TCGA-A8-A075-01 | LumB | 0 | 0.46154269 | 0 | 0.53845731 | 0 |
| TCGA-B6-A0RN-01 | LumA | 0 | 0 | 0.69076256 | 0 | 0.30923744 |
| TCGA-AO-A0JG-01 | LumA | 0 | 0 | 0.6651135 | 0 | 0.3348865 |
| TCGA-B6-A0RU-01 | Basal | 0.95765823 | 0 | 0 | 0 | 0.04234177 |
| TCGA-A1-A0SH-01 | LumA | 0 | 0 | 0.64336759 | 0 | 0.35663241 |
| TCGA-A2-A0T7-01 | LumA | 0 | 0 | 0.58795083 | 0 | 0.41204917 |
| TCGA-A2-A0T0-01 | Basal | 0.5882398 | 0.30051298 | 0 | 0.11124723 | 0 |
| TCGA-A2-A0T5-01 | LumA | 0 | 0 | 0.6045521 | 0 | 0.3954479 |
| TCGA-BH-A0RX-01 | Basal | 0.97675939 | 0.02171545 | 0 | 0.00152517 | 0 |
| TCGA-A7-A0CE-11 | Normal | 0 | 0 | 0.49254293 | 0 | 0.50745707 |
| TCGA-A2-A0SW-01 | LumB | 0 | 0.41336411 | 0 | 0.58663589 | 0 |
| TCGA-BH-A0B3-11 | Normal | 0 | 0 | 0.47183099 | 0 | 0.52816901 |
| TCGA-BH-A0BC-01 | LumA | 0 | 0 | 0.813957 | 0.17027537 | 0.01576764 |
| TCGA-A2-A0T1-01 | Her2 | 0 | 0.62939057 | 0 | 0.37060943 | 0 |
| TCGA-BH-A0DK-11 | LumA | 0 | 0 | 0.51580839 | 0 | 0.48419161 |
| TCGA-B6-A0RT-01 | Basal | 0.85172263 | 0.05608373 | 0 | 0.09219363 | 0 |
| TCGA-BH-A0BA-11 | Normal | 0 | 0 | 0.46555331 | 0 | 0.53444669 |
| TCGA-A2-A0SV-01 | LumB | 0 | 0.38972756 | 0 | 0.61027244 | 0 |
| TCGA-A1-A0SM-01 | LumA | 0 | 0.35972955 | 0.37304718 | 0.26722328 | 0 |
| TCGA-A7-A0DB-11 | LumA | 0 | 0 | 0.54766314 | 0 | 0.45233686 |
| TCGA-A2-A0SU-01 | LumA | 0 | 0 | 0.70113728 | 0 | 0.29886272 |
| TCGA-A1-A0SE-01 | LumA | 0 | 0 | 0.65584527 | 0 | 0.34415473 |
| TCGA-B6-A0RV-01 | LumA | 0 | 0 | 0.7472869 | 0 | 0.2527131 |
| TCGA-BH-A0BV-11 | Normal | 0 | 0 | 0.45103196 | 0 | 0.54896804 |
| TCGA-A7-A0CH-11 | LumA | 0 | 0 | 0.53563662 | 0 | 0.46436338 |
| TCGA-BH-A0DH-01 | LumA | 0 | 0 | 0.75554808 | 0 | 0.24445192 |
| TCGA-A1-A0SK-01 | Basal | 0.47894956 | 0.21896028 | 0 | 0.30209016 | 0 |
| TCGA-BH-A0H7-11 | Normal | 0 | 0 | 0.49103076 | 0 | 0.50896924 |
| TCGA-BH-A0DQ-01 | LumA | 0 | 0 | 0.80174306 | 0.10345286 | 0.09480407 |
| TCGA-A2-A0SY-01 | LumA | 0 | 0 | 0.76544467 | 0 | 0.23455533 |
| TCGA-BH-A0BJ-11 | Normal | 0 | 0 | 0.45068737 | 0 | 0.54931263 |
| TCGA-A2-A0ST-01 | Basal | 0.66716667 | 0 | 0 | 0 | 0.33283333 |
| TCGA-BH-A0HP-01 | LumA | 0 | 0 | 0.64924976 | 0 | 0.35075024 |
| TCGA-A2-A0EN-01 | LumA | 0 | 0 | 0.70144307 | 0 | 0.29855693 |
| TCGA-BH-A0W3-01 | LumB | 0 | 0.04639893 | 0.42895338 | 0.52464769 | 0 |
| TCGA-AN-A0XS-01 | LumA | 0 | 0 | 0.71949863 | 0 | 0.28050137 |
| TCGA-A2-A0YH-01 | LumB | 0 | 0.07998337 | 0.33626269 | 0.58375393 | 0 |
| TCGA-AR-A0U0-01 | Basal | 0.94779608 | 0.05220392 | 0 | 0 | 0 |
| TCGA-A2-A0YF-01 | LumA | 0 | 0 | 0.69673255 | 0.30326745 | 0 |
| TCGA-A2-A04R-01 | LumB | 0 | 0.03108981 | 0.29791625 | 0.67099394 | 0 |
| TCGA-AN-A0XV-01 | LumA | 0 | 0 | 0.74561688 | 0 | 0.25438312 |
| TCGA-B6-A0WY-01 | LumA | 0 | 0 | 0.55208657 | 0 | 0.44791344 |
| TCGA-AN-A0XW-01 | Her2 | 0 | 0.36333996 | 0.28413519 | 0.35252485 | 0 |
| TCGA-AN-A0XN-01 | LumA | 0 | 0 | 0.75499363 | 0 | 0.24500638 |
| TCGA-A2-A0YE-01 | Basal | 0.86235409 | 0.13764591 | 0 | 0 | 0 |
| TCGA-AR-A0U2-01 | LumB | 0 | 0.42175501 | 0 | 0.57824499 | 0 |
| TCGA-A2-A0YT-01 | LumB | 0 | 0.40290314 | 0.01831618 | 0.57878068 | 0 |
| TCGA-AR-A0TU-01 | Basal | 0.65090828 | 0.13960336 | 0 | 0.20948836 | 0 |
| TCGA-B6-A0WV-01 | LumB | 0 | 0.07394058 | 0.38373113 | 0.5423283 | 0 |
| TCGA-BH-A0WA-01 | Basal | 0.90911884 | 0 | 0 | 0 | 0.09088116 |
| TCGA-A2-A0YC-01 | LumA | 0 | 0 | 0.75907046 | 0.22105143 | 0.01987811 |
| TCGA-AN-A0XT-01 | LumA | 0 | 0 | 0.67961877 | 0 | 0.32038123 |
| TCGA-AN-A0XO-01 | LumA | 0 | 0 | 0.7352006 | 0 | 0.2647994 |
| TCGA-BH-A0W4-01 | LumA | 0 | 0 | 0.65339494 | 0 | 0.34660506 |
| TCGA-B6-A0X5-01 | LumB | 0 | 0.33372479 | 0 | 0.66627521 | 0 |
| TCGA-AN-A0XU-01 | Basal | 0.95165037 | 0 | 0 | 0 | 0.04834963 |
| TCGA-B6-A0WX-01 | Normal | 0.39473528 | 0 | 0.0769505 | 0 | 0.52831422 |
| TCGA-AN-A0XP-01 | LumA | 0 | 0 | 0.77336707 | 0 | 0.22663294 |
| TCGA-A2-A0YJ-01 | Basal | 0.74285167 | 0 | 0 | 0 | 0.25714833 |
| TCGA-A2-A0YM-01 | Basal | 0.87688122 | 0.12311878 | 0 | 0 | 0 |
| TCGA-B6-A0X1-01 | Basal | 0.53443801 | 0.2251281 | 0 | 0.24043389 | 0 |
| TCGA-BH-A0W5-01 | LumA | 0 | 0 | 0.64693945 | 0 | 0.35306055 |
| TCGA-B6-A0X4-01 | LumA | 0 | 0 | 0.63935446 | 0 | 0.36064554 |
| TCGA-A2-A0YK-01 | LumA | 0 | 0 | 0.53832461 | 0 | 0.46167539 |
| TCGA-A2-A0YD-01 | LumA | 0 | 0 | 0.69869246 | 0 | 0.30130754 |
| TCGA-A2-A0YL-01 | LumA | 0 | 0 | 0.59485824 | 0 | 0.40514176 |
| TCGA-A2-A0YG-01 | LumB | 0 | 0.47096295 | 0 | 0.52903705 | 0 |
| TCGA-B6-A0WT-01 | LumA | 0 | 0 | 0.81144494 | 0.06526906 | 0.123286 |
| TCGA-B6-A0WZ-01 | LumA | 0 | 0.06926437 | 0.80253761 | 0.12819802 | 0 |
| TCGA-AR-A0U4-01 | Basal | 0.91674386 | 0 | 0 | 0.08325614 | 0 |
| TCGA-AN-A0XR-01 | LumB | 0 | 0.18232919 | 0.13688544 | 0.68078537 | 0 |
| TCGA-B6-A0WW-01 | LumA | 0 | 0.0329592 | 0.65009799 | 0.31694281 | 0 |
| TCGA-AR-A0U3-01 | LumB | 0 | 0.06037008 | 0.35971994 | 0.57990998 | 0 |
| TCGA-AQ-A04L-01 | LumA | 0 | 0.00173347 | 0.68282818 | 0 | 0.31543836 |
| TCGA-AO-A12E-01 | LumA | 0 | 0 | 0.66218671 | 0.02070469 | 0.31710861 |
| TCGA-E2-A105-01 | LumA | 0 | 0 | 0.72229351 | 0.26872425 | 0.00898224 |
| TCGA-AO-A03U-01 | Normal | 0 | 0 | 0.46192873 | 0 | 0.53807128 |
| TCGA-E2-A10B-01 | LumA | 0 | 0 | 0.70957964 | 0.26577066 | 0.0246497 |
| TCGA-E2-A10E-01 | LumA | 0 | 0 | 0.75361504 | 0.04900107 | 0.19738388 |
| TCGA-E2-A10C-01 | LumA | 0 | 0.10915701 | 0.51196415 | 0.37887884 | 0 |
| TCGA-AO-A129-01 | Basal | 1 | 0 | 0 | 0 | 0 |
| TCGA-AO-A125-01 | LumA | 0 | 0 | 0.66778274 | 0 | 0.33221726 |
| TCGA-E2-A107-01 | LumB | 0 | 0.21579994 | 0.15771526 | 0.6264848 | 0 |
| TCGA-AO-A03M-01 | Her2 | 0 | 0.44775547 | 0.3213966 | 0.23084794 | 0 |
| TCGA-AO-A12G-01 | LumA | 0 | 0 | 0.74235695 | 0 | 0.25764305 |
| TCGA-B6-A0X7-01 | LumA | 0 | 0 | 0.6739163 | 0 | 0.3260837 |
| TCGA-AO-A124-01 | Basal | 0.91419723 | 0 | 0 | 0 | 0.08580277 |
| TCGA-E2-A10F-01 | LumA | 0 | 0 | 0.74913251 | 0 | 0.25086749 |
| TCGA-AO-A03N-01 | LumA | 0 | 0.34210724 | 0.36496954 | 0.29292322 | 0 |
| TCGA-A2-A0YI-01 | LumA | 0 | 0 | 0.65326496 | 0 | 0.34673505 |
| TCGA-AO-A126-01 | LumA | 0 | 0 | 0.82033393 | 0 | 0.17966607 |
| TCGA-AN-A0XL-01 | LumA | 0 | 0 | 0.64764278 | 0 | 0.35235722 |
| TCGA-AQ-A04H-01 | LumB | 0 | 0.20734671 | 0.23605538 | 0.55659791 | 0 |
| TCGA-E2-A106-01 | LumA | 0 | 0 | 0.67005141 | 0 | 0.32994859 |
| TCGA-AO-A12C-01 | LumA | 0 | 0 | 0.65946008 | 0 | 0.34053992 |
| TCGA-E2-A108-01 | LumA | 0 | 0 | 0.55215291 | 0 | 0.44784709 |
| TCGA-BH-A0B2-01 | LumA | 0 | 0 | 0.6798649 | 0 | 0.3201351 |
| TCGA-AO-A12B-01 | LumA | 0 | 0 | 0.68915152 | 0.31084849 | 0 |
| TCGA-BH-A0B2-11 | Normal | 0 | 0 | 0.46189257 | 0 | 0.53810744 |
| TCGA-E2-A109-01 | LumB | 0 | 0.3919505 | 0 | 0.6080495 | 0 |
| TCGA-AO-A128-01 | Basal | 0.62368948 | 0.22352941 | 0 | 0.15278111 | 0 |
